# The role of mesenchymal cells in cholangiocarcinoma

**DOI:** 10.1242/dmm.050716

**Published:** 2024-12-13

**Authors:** Mireia Sueca-Comes, Elena Cristina Rusu, Jennifer C. Ashworth, Pamela Collier, Catherine Probert, Alison Ritchie, Marian Meakin, Nigel P. Mongan, Isioma U. Egbuniwe, Jesper Bøje Andersen, David O. Bates, Anna M. Grabowska

**Affiliations:** ^1^ Translational Medical Science, School of Medicine, Biodiscovery Institute, University of Nottingham, Nottingham NG7 2RD, UK; ^2^ Institute of Integrative Systems Biology (I2Sysbio), University of Valencia and Consejo Superior de Investigaciones Científicas (CSIC), 46980 Valencia, Spain; ^3^ School of Veterinary Medicine and Science, Sutton Bonington Campus, University of Nottingham, Leicestershire LE12 5RD, UK; ^4^ Biotech Research and Innovation Centre (BRIC), Department of Health and Medical Sciences, University of Copenhagen, Copenhagen DK-2200, Denmark; ^5^ Department of Pharmacology, Weill Cornell Medicine, New York, NY 10065, USA

**Keywords:** Cholangiocarcinoma, PDX models, Mesenchymal stem cells, Signalling pathways, Tumour microenvironment

## Abstract

The tumour microenvironment (TME) significantly influences tumour formation and progression through dynamic interactions. Cholangiocarcinoma (CCA), a highly desmoplastic tumour, lacks early diagnostic biomarkers and has limited effective treatments owing to incomplete understanding of its molecular pathogenesis. Investigating the role of the TME in CCA progression could lead to better therapies. RNA sequencing was performed on seven CCA patient-derived xenografts (PDXs) and their corresponding patient samples. Differential expression analysis was conducted, and Qiagen Ingenuity Pathway Analysis was used to predict dysregulated pathways and upstream regulators. PDX- and cell line-derived spheroids, with and without immortalised mesenchymal stem cells, were grown and analysed for morphology, growth and viability. Histological analysis confirmed biliary phenotypes. RNA sequencing indicated upregulation of extracellular matrix-receptor interaction and PI3K-AKT pathways in the presence of mesenchymal cells, with several genes linked to poor survival. Mesenchymal cells restored the activity of inhibited cancer-associated kinases. Thus, adding mesenchymal cells to CCA spheroid models restored key paracrine signalling pathways lost in PDXs, enhancing tumour growth and viability. These findings highlight the importance of including stromal components in cancer models to improve pre-clinical studies.

## Teaser

**Summary:** Exploring the tumour microenvironment in cholangiocarcinoma enables key pathways to be restored in pre-clinical models, enhancing their accuracy and application in understanding of cancer progression.

## INTRODUCTION

Cholangiocarcinoma (CCA) is a highly malignant tumour arising from the biliary epithelium, characterised by its aggressive behaviour and poor prognosis (Razumilava and Gores, 2014). Traditional two-dimensional (2D) cell culture systems have been extensively used to study CCA, but they often fail to recapitulate the complex architecture and microenvironment of tumours, because they lack multi-dimensional cell-cell interactions and, usually, molecular and cellular stromal elements, resulting in limited translational relevance (Antoni et al., 2015). In recent years, three-dimensional (3D) cell culture models, have emerged as valuable tools for investigating the biology and therapeutic responses of CCA (Mikhail et al., 2013; Gilazieva et al., 2020). However, although, compared to traditional 2D models, 3D models, such as spheroids and organoids, better reflect the heterogeneity and complexity of solid tumours, making them more physiologically relevant for studying tumour behaviour and drug responses (Edmondson et al., 2014; Vinci et al., 2012), such models still lack stromal cells (Gilazieva et al., 2020).

CCA is histologically characterised as a highly desmoplastic tumour. The abundant fibrotic stroma that surrounds and infiltrates the tumour is also known to modulate the progression and invasiveness of CCA (Fabris et al., 2019; Sirica and Gores, 2014; Affo et al., 2021). The CCA stroma consists of cancer-associated fibroblasts (CAFs), endothelial and lymphatic cells, and a complex collection of inflammatory cells (macrophages, natural killer cells, neutrophils and T cells) (Banales et al., 2020). In addition to this, the tumour stroma also contains an extensive network of extracellular matrix (ECM) proteins (laminin, collagens, and fibronectin) (Govaere et al., 2016; Szendroi and Lapis, 1985).

Studies have shown that CAFs can originate from bone marrow-derived circulating mesenchymal stem cells (MSCs) (Russo et al., 2006), liver-resident hematopoietic stem cells (Okabe et al., 2009) and/or portal fibroblasts (Dranoff and Wells, 2010). They are characterised by the expression of several markers, the most common ones being α-SMA (also known as ACTA2), the mucin-like transmembrane glycoprotein podoplanin and the cell surface metalloprotease CD10 (also known as MME). CAFs are the major component in the CCA tumour microenvironment (TME), and their presence correlates with poor patient survival (Chuaysri et al., 2009). CAFs, in the context of intrahepatic CCA, are a hugely heterogeneous population displaying distinctive phenotypic traits (Fabris et al., 2020; Affo et al., 2021).

CAFs can shape the TME, as well as influence tumour growth and invasion, through releasing pro-oncogenic paracrine mediators. These include TGF-β1, hepatocyte growth factor (HGF), EGF, SDF-1 (also known as CXCL12), connective tissue growth factor (CTGF; also known as CCN2), ECM components and matrix metalloproteinases (Sirica, 2011). One of the most significant signalling pathways between CCA cells and CAFs is the HBEGF/EGFR signalling pathway, in which there is an intense two-way communication by which CCA cells activate CAFs and, in turn, CAFs sustain the invasive phenotype of cancer cells; activation of EGFR triggers TGF-β1 production by CCA cells, further enhancing fibroblast activation and CAF synthesis of HBEGF (Claperon et al., 2013).

When modelling cancer with an aim of understanding the underlying biology, identifying potential therapeutic targets and screening new therapeutic agents, molecular heterogeneity and the complexity of the stromal influence should be taken into consideration (Affo et al., 2021; Song et al., 2022; Zhang et al., 2020). However, standard 2D models based on such cells are missing the influence of the TME and likely have adapted to growth on plastic in nutrient-rich conditions. Organoid models, although involving growth in a 3D setting directly derived from patient samples, lack other aspects of the TME, except where provided through use of complex media e.g. in the form of growth factors (van de Wetering et al., 2015). Patient-derived xenograft (PDX) models, established by directly expanding patient tissue in an *in vivo* setting, theoretically maintain a more complex TME and provide a more clinically relevant model (Hidalgo et al., 2014). However, in common with other xenograft models, they are grown in immunodeficient mice and thus lack many of the immune components of the TME (Kopetz et al., 2012). Additionally, it has become apparent that the human stroma, transplanted along with the human cancer cells, is rapidly replaced by mouse stroma (Isella et al., 2015; Jones et al., 2023). Loss of human stromal cells in such models potentially results in a disconnect whereby mouse-human interactions are not able to take place across the species barrier.

Therefore, this study aims first to identify signalling pathways that are lost in xenograft models in the absence of human stromal signalling and, second, to examine, in CCA spheroid models derived from both cell lines and PDXs, whether addition of MSCs can restore such pathways. MSCs have recently gained attention for their role in tumour growth and progression (Spaeth et al., 2009; Pal et al., 2020). They are multipotent stromal cells that are recruited by tumours and can be derived from various sources, including bone marrow, adipose tissue and umbilical cord (Bieback and Brinkmann, 2010). These cells possess immunomodulatory properties and can interact with tumour cells, influencing many aspects of tumour biology, such as proliferation, angiogenesis and the TME (Hoogduijn et al., 2013). Although the impact of MSCs on CCA remains largely unexplored, studies in other cancer types have demonstrated their pro-tumorigenic effects following activation to become CAFs (Kidd et al., 2009; Katsuda et al., 2013).

The findings from this study have the potential to enhance our understanding of the tumour-stroma crosstalk in CCA and provide insights into the roles of MSCs in this disease. The identification of upregulated genes, enriched pathways and phenotypic characteristics of the spheroids may contribute to the development of personalised treatment approaches for CCA patients.

## RESULTS

### Identification of lost signalling pathways in PDX models and restoration via MSC addition in CCA spheroid models

**Fig. 1.**
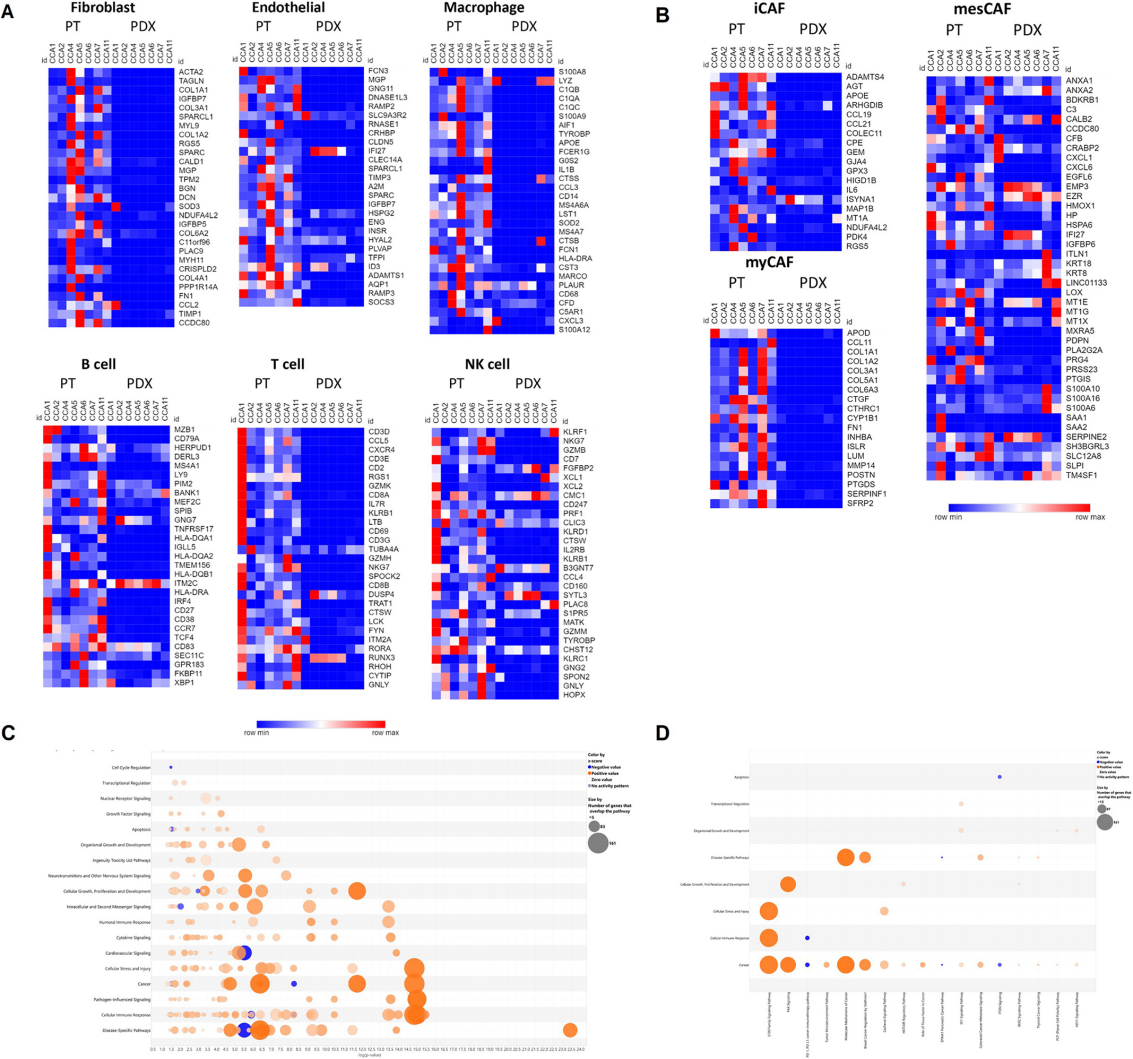
**Loss of human stromal cells and dysregulation of cancer-associated pathways in CCA PDXs.** (A,B) Heatmaps of genes identified as being associated with different populations of stromal cells (A) or cancer-associated fibroblasts (B) through single-cell analysis (Song et al., 2022; Affo et al., 2021). Blue and red indicate downregulated and upregulated genes, respectively. (C,D) Bubble charts showing canonical pathways associated with signalling (C) or with cancer (D) that were significantly dysregulated in patient tissues compared to PDXs. Orange and blue bubbles indicate activated and inhibited pathways, respectively; the size of the bubble indicates the number of genes in each gene set. CCA, cholangiocarcinoma; iCAF, immune-related cancer-associated fibroblast (CAF); myCAF, myofibroblast-like CAF; mesCAF, mesenchymal CAF; NK, natural killer; PDX, patient-derived xenograft; PT, patient tissue.

Whole-genome expression profiling was used to compare the human transcriptome of a panel of seven CCA PDXs with that of the patient samples from which they were derived. Analysis of expression of markers of a variety of stromal populations (CAFs, endothelial and immune cells) derived from single-cell expression analysis of CCA tumours (Zhang et al., 2020) showed that human stromal cells were lost in the PDXs (Fig. 1A), with the majority of markers at lower levels in the PDXs than in their respective patient samples. This extended to markers of CCA CAF subtypes (Affo et al., 2021), which were also mainly absent in the PDXs (Fig. 1B).

Because paracrine signalling from stromal cells is known to drive activation of specific pathways in cancer cells, and, given the potential for complete loss of some stromal cells in immunodeficient mouse models and/or species disconnect even where human stromal cells are replaced by mouse stromal cells, the possibility that some cancer-associated pathways are dysregulated in the PDXs was investigated.

First, human genes differentially expressed in the patient samples and PDXs were identified; then, based on this gene set, the canonical pathways predicted to be differentially active in the patient and PDX samples were examined using Qiagen Ingenuity Pathway Analysis (IPA). A broad set of signalling pathways was apparently affected (Fig. 1C), including pathways associated with cancer (Fig. 1D; Table S1).

Using IPA Upstream Regulator Analysis, a wide range of upstream regulators was identified, including growth factors, cytokines, kinases and transcription regulators (Table S2), predicted to be driving signalling pathways dysregulated in the PDXs. Considering kinases as druggable targets and the rising use of PDX models for evaluating kinase inhibitors as potential anti-cancer agents, kinase-associated pathways were the focal point in the next stage of the analysis.

**Fig. 2.**
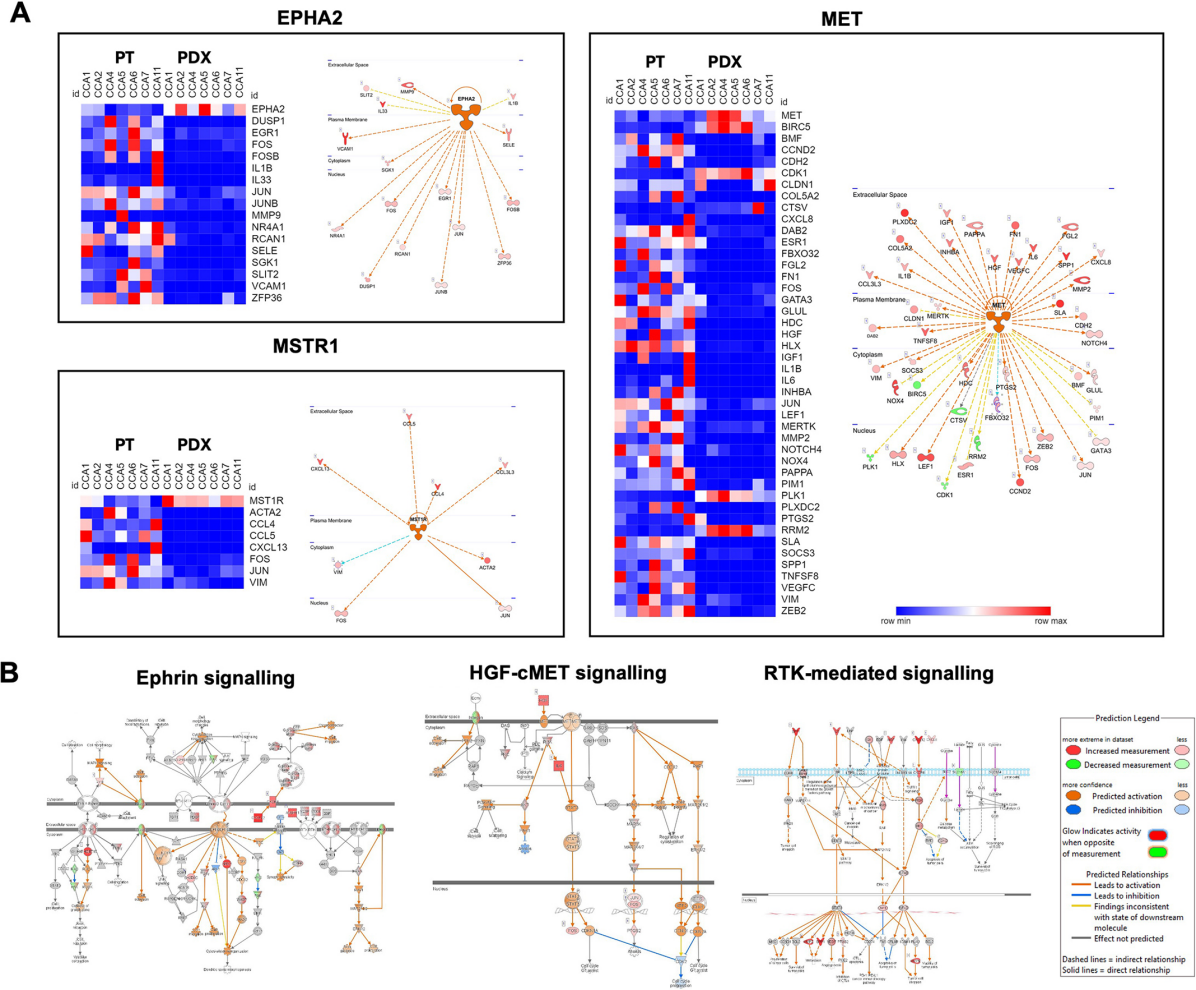
**Dysregulation of paracrine signalling pathways in cancer cells within CCA PDXs.** (A) Heatmaps of genes involved in the pathways downstream of the top 3 inhibited cancer-associated kinases (ICAKs; kinases predicted to be inhibited in the PDXs in spite of maintained expression) – MET, EPHA2 and MSTR1 – and diagrams of the respective networks. Blue and red indicate downregulated and upregulated genes, respectively. (B) Diagrams of three pathways involving these and other kinases. Pathways are provided by Qiagen Ingenuity Pathway Analysis and were overlaid with information to demonstrate molecules that were overexpressed or underexpressed and/or predicted to be activated/inhibited in the patient tissues compared to the PDXs.

Some of the pathways apparently active in the patient samples and not in the PDXs included stromal signalling pathways that are not truly absent (e.g. signalling pathways associated with innate immune cells or endothelial cells) but present as mouse genes derived from mouse stromal cells, not considered in our analysis of human gene expression. Thus, in order to identify paracrine signalling pathways dysregulated more specifically in the cancer cells within the PDXs, the focus was directed to a subset of kinases, the expression of which was maintained or apparently enriched in the PDXs (suggesting that they are present in the human cancer cell compartment, rather than in the mouse stromal compartment), but the associated downstream pathway of which was predicted to be activated in the patient samples compared with the PDXs (based on activation *z*-score >2), i.e. inhibited in the PDXs, designated as inhibited cancer-associated kinases (ICAKs) (Table S3). Such a situation could arise because, although the kinase is present, upstream events that would normally activate them are missing, as would be the case if a paracrine signalling molecule were absent or unable to bind to its cognate receptor. Heatmaps of the downstream components of the pathways driven by the top 3 of these ICAKs confirmed that signalling in these pathways was attenuated in the PDXs (Fig. 2A). Loss of such signalling potentially affects cancer-associated functional characteristics, such as proliferation, survival, adhesion, migration, survival and invasion (Fig. 2B).

**Fig. 3.**
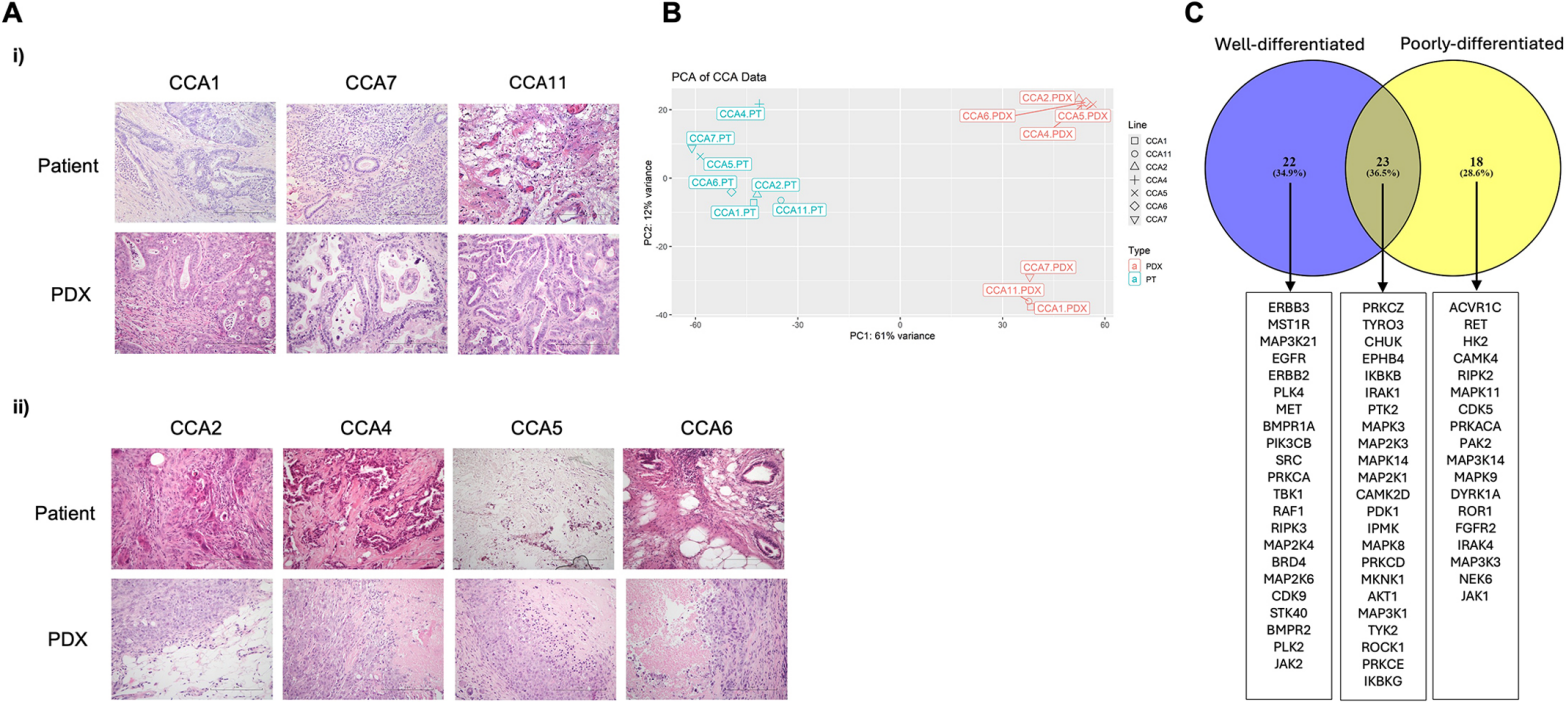
**Classification and dysregulation of upstream regulators in CCA PDXs.** Histology and Upstream Regulator Analysis for two PDX subtypes. (A) Haematoxylin and Eosin (H&E) staining of patient and PDX tissues for two subtypes of PDX: well-differentiated (i) and poorly differentiated (ii). Objective, 20×. Scale bars: 200[Supplementary-material sup1]μm. (B) Principal component analysis plot based on gene expression in patient and PDX samples, indicating two clusters of PDXs. PC, principal component. (C) Venn diagram to illustrate ICAKs found to be common or unique to the two PDX subtypes.

Based on the histology of the PDXs (Fig. 3A) and principal component analysis (Fig. 3B), it was observed that the PDXs fell into two categories: well-differentiated (WD) PDXs (Fig. 3Ai), with histology resembling those of the original patient tissues, and more poorly differentiated (PD) tumours (Fig. 3Aii). Because there was potential for these two categories of PDX to be driven by different signalling pathways, analysis of upstream regulators for each separately was additionally carried out, and ICAKs were identified (Tables S4 and S5). Interestingly, although there are common ICAKs dysregulated in both the WD- and PD-PDXs, there are ICAKs uniquely dysregulated in each type of PDX (Fig. 3C).

In order to investigate the potential for restoring such signalling pathways, 3D spheroid co-culture models were established involving addition of immortalised bone marrow-derived MSCs (iMSCs) to CCA cells. The rationale for use of MSCs is that these multipotent cells have the potential to become activated to CAFs and provide human paracrine signals to restore those lost in xenografts and in standard mono-culture models (Kidd et al., 2009; Katsuda et al., 2013).

### Viability and growth dynamics of CCA spheroids in mono- and co-culture with iMSCs

**Fig. 4.**
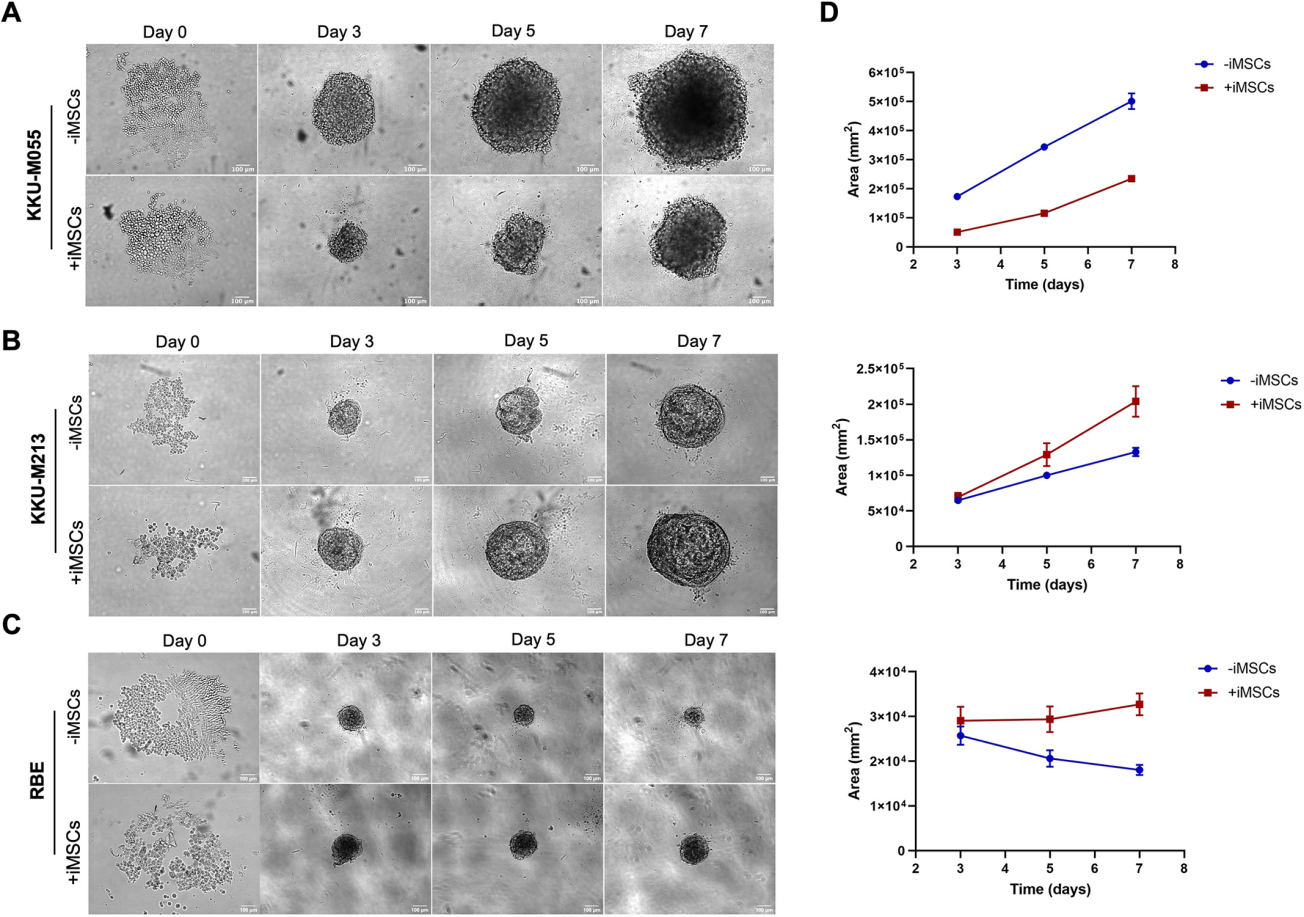
**Effects of iMSCs on CCA cells in spheroid co-culture.** (A-C) CCA cells [KKU-M055 (A), KKU-M213 (B) and RBE (C)] were cultured as spheroids alone (mono-culture) or iMSCs at a 1:2 ratio (cancer cells:iMSCs) (co-culture) in an ultra-low attachment (ULA) 96-well round-bottom plate at a final cell density of 1000 cells per well with 1001[Supplementary-material sup1]μg/ml basement membrane extract (BME). The effect of co­ culture with iMSCs was assessed using brightfield microscopy at day 0, 3, 5 and 7 at 10× magnification. Representative spheroids are shown. Scale bars: 100[Supplementary-material sup1]μm. The spheroids were analysed after initiation to determine area based on image analysis of brightfield micrographs captured at each timepoint from three independent replicates (mean±s.e.m.). iMSC, immortalised mesenchymal stem cell.

Spheroids of three CCA cell lines – KKU-M055, KKU-M213 and RBE – were established as mono-cultures (CCA cells only) or as co-cultures with the addition of iMSCs at a 1:2 ratio (cancer cells:iMSCs). The morphology of the spheroids formed from different cell lines exhibited distinct characteristics (Fig. 4). Upon initial seeding, individual cells were observed. However, after 3 days, spheroids were formed by all three cell lines. The KKU-M055 line formed loosely packed spheroids, and the addition of iMSCs resulted in the formation of more compact spheroids (Fig. 4A). In contrast, the KKU-M213 line formed spherical spheroids with well-defined edges, and this morphology was further enhanced by the presence of iMSCs (Fig. 4B). The RBE cell line produced the smallest spheroids, which, in the absence of iMSCs, became smaller over time (Fig. 4C). To further support these observations, the area of the spheroids was measured from the brightfield images at each timepoint. For the KKU-M055 and KKU-M213 lines, there was an increase in the area of both mono- and co-cultures over time. However, the addition of iMSCs to the RBE-derived spheroids led to an increased area, maintained over time (Fig. 4D), which may reflect improved cell viability or formation of looser spheroids.

**Fig. 5.**
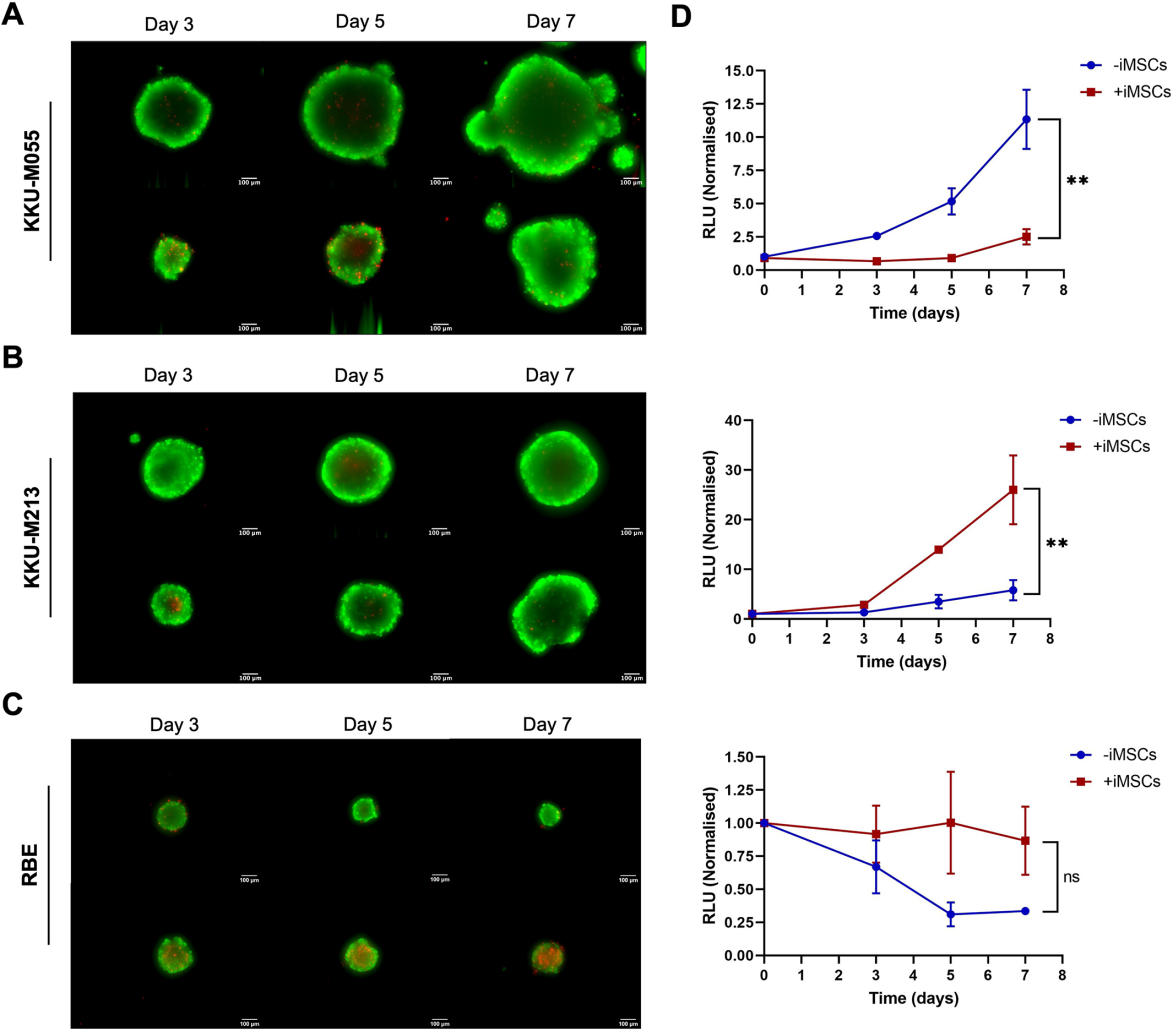
**Cell viability of CCA spheroids as mono-cultures and iMSC co-cultures.** (A-C) CCA cells [KKU-M055 (A), KKU-M213 (B) and RBE (C)] were cultured as spheroids alone (mono-culture) or iMSCs at a 1:2 ratio (cancer cells:iMSCs) (co-culture) in a ULA 96-well round-bottom plate at a final cell density of 1000 cells per well with 100[Supplementary-material sup1]μg/ml BME. Live/dead cell staining was carried out using calcein acetoxymethyl (green, live cells) and ethidium homodimer (red, dead cells) at days 3, 5 and 7. Representative spheroids are shown. Scale bars: 100[Supplementary-material sup1]μm. Progressive growth of the spheroid was monitored by adding D-luciferin at day 0, 3, 5 and 7. Values were normalised to day 0 (mean±s.e.m.). Co-cultures were compared to the mono-culture via paired two-tailed Student's *t*-test (*n*=2). ns, not significant (*P*>0.05); **P<0.01. RLU, relative luminescence units.

Next, the viability of the spheroids was assessed. The live/dead staining results confirmed the overall viability of the cells (Fig. 5A-C). In both mono- and co-culture spheroids of all cell lines, more than 95% of the cells were viable, except for the RBE cells (Fig. 5C), for which the co-cultures with iMSCs exhibited higher ethidium homodimer (EthD-1) staining, indicating increased cell death, indicating that larger spheroids do not necessarily reflect higher cell viability.

However, because the live/dead staining measurements cannot differentiate between signals derived from cancer cells and iMSCs, a more direct approach was adopted to measure cell viability specifically in the cancer cells. Cancer cells were transduced with a lentivirus to introduce the firefly luciferase gene, resulting in only the cancer cells emitting light upon addition of the luciferase substrate. The emitted light intensity at each timepoint served as a reflection of the number of viable cancer cells present. Results from the luminescent assay revealed that the growth of KKU-M213 cells as spheroids was significantly dependent on the addition of iMSCs (*P*=0.016, *n*=3). Conversely, the growth of KKU-M055 cells was higher in mono-culture spheroids (*P*=0.002, *n*=3). Interestingly, although viability of cancer cells was maintained over time in the RBE co-culture spheroids, there was no significant difference in growth between mono- and co-culture spheroids (Fig. 5D).

### Morphological and phenotypic characterisation of CCA spheroids in mono- and co-culture with iMSCs

To examine the detailed morphology of the CCA spheroids, paraffin-embedded sections of microarrayed CCA mono- and iMSCs co-culture spheroids were subjected to Haematoxylin and Eosin (H&E) staining. Furthermore, immunohistochemistry was employed to confirm the biliary phenotype of the cells within the spheroids using CK7 (also known as KRT7) and CK19 (also known as KRT19) as markers. Vimentin staining was used to localise the iMSCs, and Ki-67 (also known as MKI67) staining was performed to assess proliferation. For the RBE cells, some staining for mono-culture spheroids is missing owing to the smaller size of the spheroids compared to the others, preventing a meaningful comparison between mono- and co-culture.

**Fig. 6.**
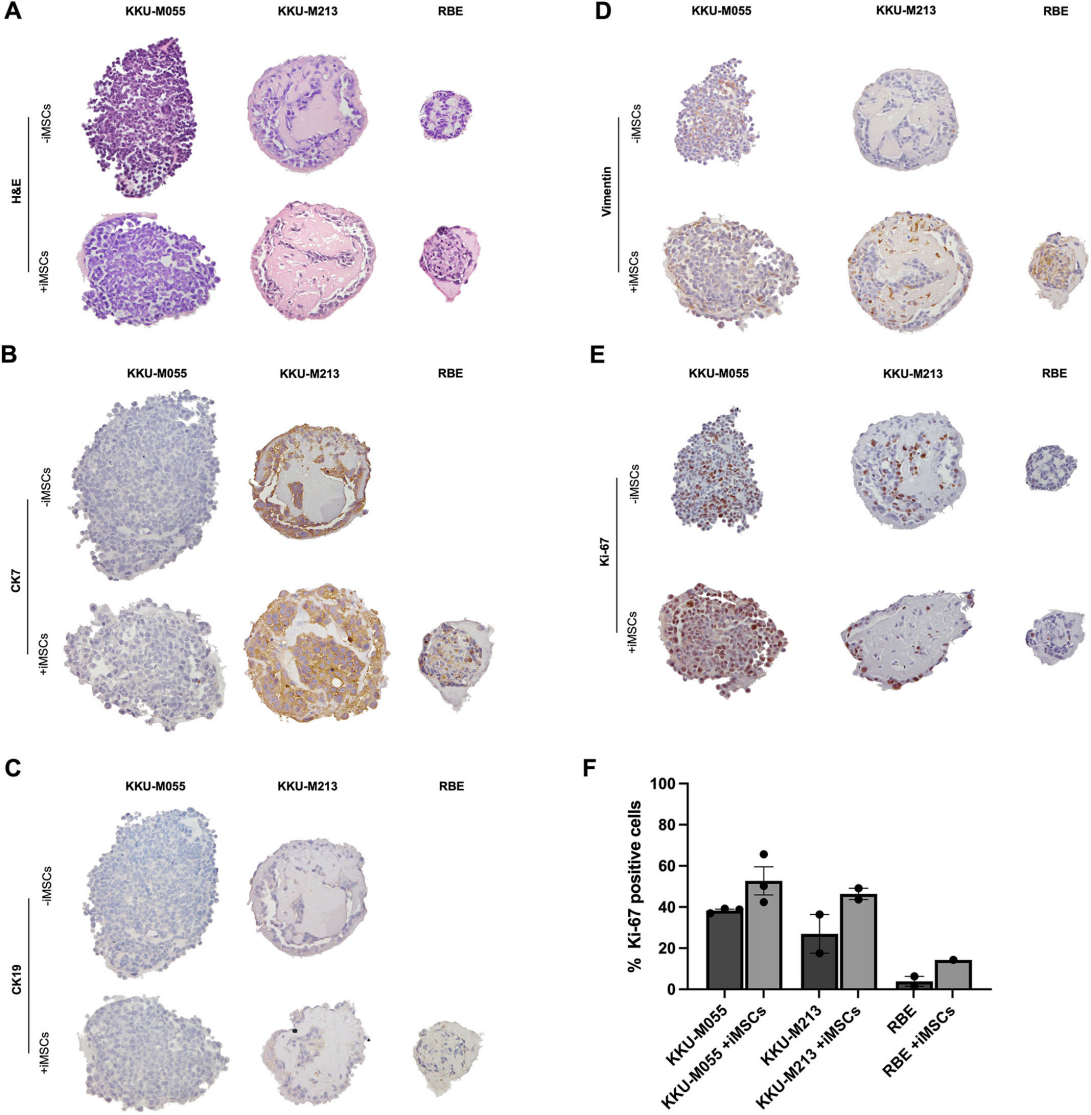
**Morphology and protein expression of CCA spheroids.** (A-E) CCA cells (KKU-M055, KKU-M213 and RBE) were cultured as spheroids alone (mono-culture) or iMSCs at a 1:2 (cancer cells:iMSCs) ratio (co-culture) in a ULA 96-well round-bottom plate at a final cell density of 1000 cells per well with 100[Supplementary-material sup1]µg/ml BME. The spheroids were fixed at day 5 of culture, paraffin embedded, sectioned and stained with Haematoxylin and Eosin (H&E) (A), or stained for CK7 (B), CK19 (C), vimentin (D) and Ki-67 (E). (F) Ki-67 expression was quantified by manually counting the positive cells and cancer cells and calculating the ratio of positively stained cancer cells over the total of cancer cells. At least six spheroids were embedded in the array from each condition. Representative spheroids are shown at 20× magnification.

Interestingly, the presence of ductal-like structures, similar to those in CCA patient tumours, was observed within the spheroids, particularly for the KKU-M213 and RBE models (Fig. 6A). The biliary phenotype was confirmed by positive expression of CK7 and CK19, except for the KKU-M055 line (Fig. 6B,C). In the case of KKU-M213, vimentin staining was detected in the co-culture derived from this cell line, but absent in the mono-culture. Conversely, for the KKU-M055 line, positive staining for vimentin was observed in both co-culture and mono-culture (Fig. 6D). In the mono-culture spheroids, only a minor population of Ki-67-positive cells was evident. However, in the co-cultures, a larger number of brightly stained Ki-67-positive nuclei was detected. RBE cells were the exception, in which no Ki-67-positive cells were observed (Fig. 6E). Quantification further supported these findings (Fig. 6F), suggesting that iMSCs drive proliferation of the CCA cells.

### Investigation of the crosstalk and proliferation mechanism induced by iMSCs in CCA cells in co-culture

**Fig. 7.**
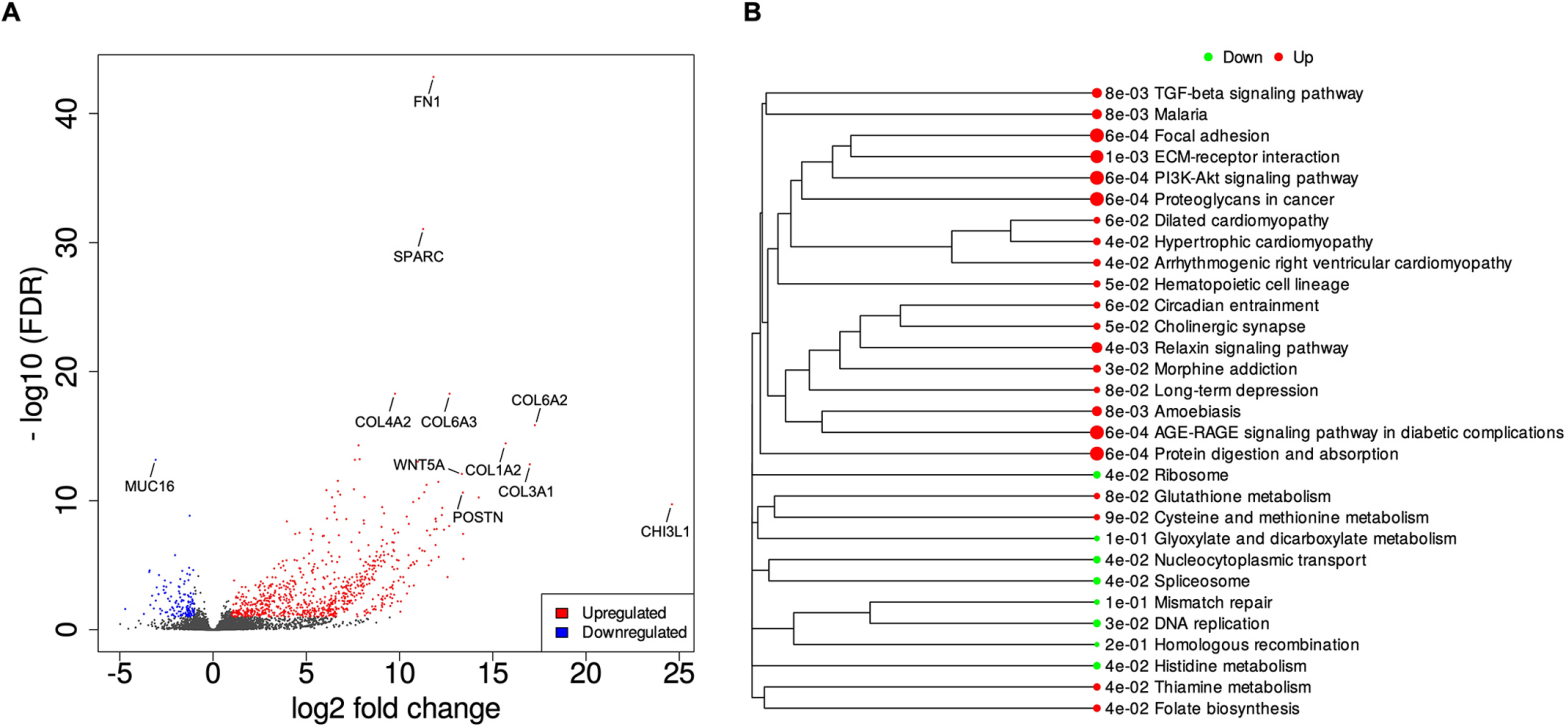
**Differential expression analysis using DESeq2 for the KKU-M213-derived spheroids.** (A) The differentially expressed genes between mono-culture and iMSCs co-culture were separated based on their false discovery rate (FDR) and fold change in a volcano plot. The upregulated and downregulated genes are represented in red and blue, respectively. (B) A gene set enrichment analysis (GSEA) was performed, and Kyoto Encyclopedia of Genes and Genomes (KEGG)-enrichment plots of representative gene sets from activated pathway are shown. The names of KEGG terms are listed, and the downregulated and upregulated pathways are represented in green and red, respectively. The length of the horizontal graph represents the gene ratio. The area of circle in the graph represents the fold-change value. These graphs were generated using integrated differential expression and pathway analysis (iDEP).

Based on RNA-sequencing (RNAseq) analysis, the top upregulated genes in the co-cultures compared with the mono-cultures, ranked by the absolute values of fold change, were shown to be *FN1*, *SPARC*, several members of the COL family, *WNT5A*, *POSTN* and *CHI3L1* (Fig. 7A).

Additionally, Kyoto Encyclopedia of Genes and Genomes (KEGG) pathway enrichment analysis was performed to identify the biological pathways that were enriched in the co-culture conditions. Among the upregulated pathways, ECM-receptor interaction [false discovery rate (FDR)=5.8×10^−4^] emerged as the most significantly enriched pathway. Notably, the PI3K-AKT signalling pathway (FDR=5.8×10^−4^) contained the highest number of upregulated genes among these pathways (Fig. 7B).

Cox proportional regression analysis for multiple genes was performed using dataset GSE89749 (Jusakul et al., 2017), with selection of the subset of samples from patients (*n*=31) with anatomic subtype, intrahepatic and fluke status matching the characteristics of the KKU-M213 line. The relationship between expression of the genes differentially expressed in the co-culture model (Table S6) and overall survival was investigated, and Kaplan–Meier plots were drawn (Fig. S1). The analysis showed that eight genes among the differentially expressed genes in the co-cultures compared with the mono-culture were significantly associated with overall survival (Bonferroni corrected *P*-value<0.05); for six of these [*ANGPTL4*, *C16ORF45* (also known as *BMERB1*), *VSTM2L*, *SERPINE2*, *CAPRIN2* and *SPOCD1*], which were upregulated in the co-cultures, high expression was associated with poor survival (Table S7).

**Fig. 8.**
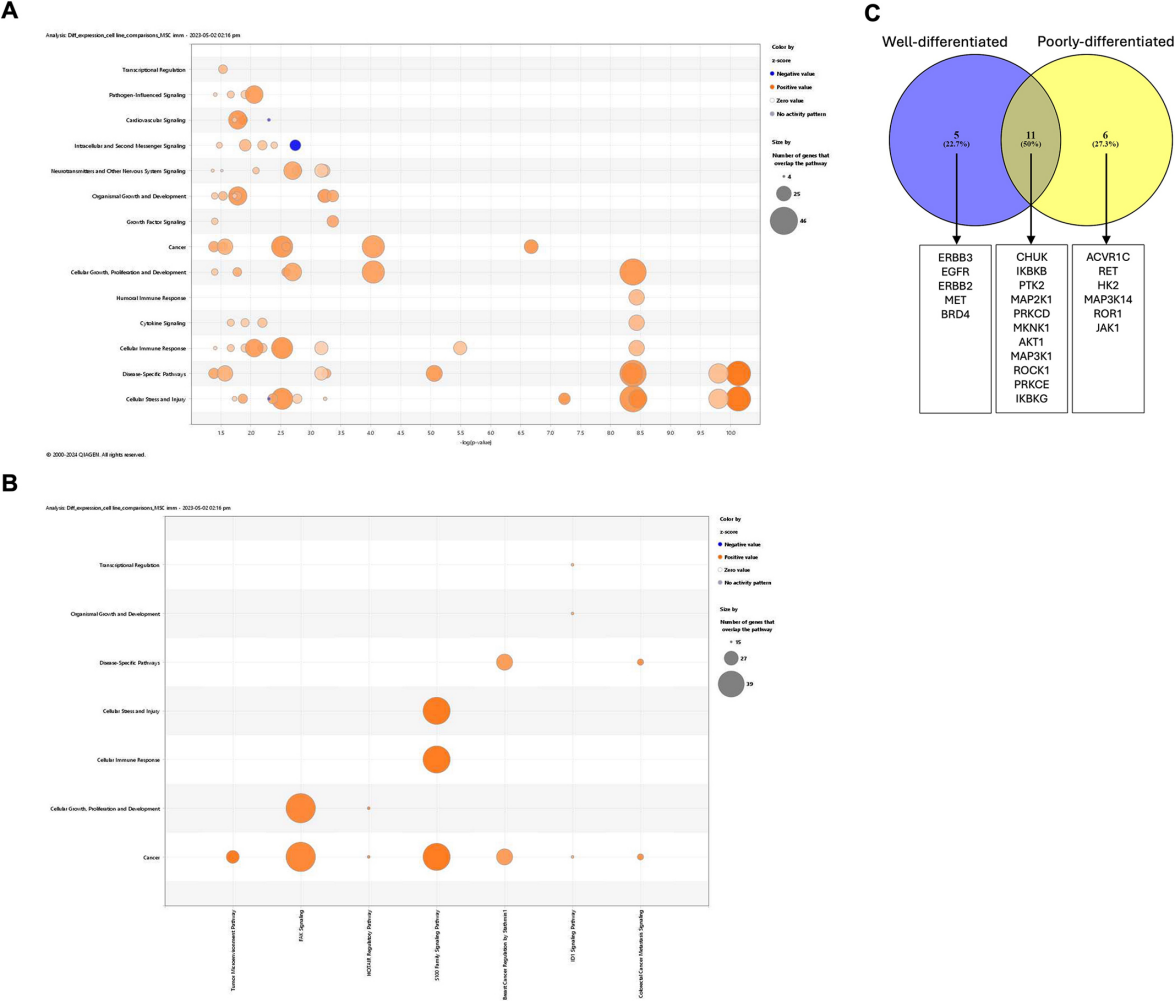
**Activation of ICAKs in MSC co-cultures and restoration of POX-type specific signalling pathways.** (A,B) Bubble charts showing canonical pathways associated with signalling (A) or with cancer (B) that were significantly activated in co-culture compared with mono-culture spheroids. Orange and blue bubbles indicate activated and inhibited pathways, respectively; the size of the bubble indicates the number of genes in each gene set. (C) Venn diagram to indicate inhibited cancer-associated kinases (ICAKs) common to or unique to well-differentiated or poorly differentiated PDXs, which were predicted to be activated in the co-culture compared to the mono-culture spheroids.

Such genes may be derived from the MSCs in the co-cultures or from the cancer cells. Because the genes are present in patient samples and suggest a microenvironment within the co-cultures that is closer to that present in the patient, further analysis to identify paracrine signals activated in the cancer cells within the co-cultures was carried out. Using a similar approach to that taken when comparing patient and PDX samples, IPA was conducted. A range of signalling pathways (Fig. 8A), including cancer-associated pathways (Fig. 8B), was shown to be altered in the co-cultures, with most pathways being activated. Upstream Regulator Analysis was again used to identify kinases that were potential upstream regulators of the changes observed in the co-culture models and overlap with the ICAKs investigated. Sixteen of the ICAKs, identified when analysing the PDXs as a whole, were activated in the MSC co-cultures (Table S8), suggesting that the MSCs have the potential to activate paracrine signal pathways lost when human stromal cells are replaced by mouse stromal cells in xenografts. The potential for MSCs to restore signalling pathways specific to the two types of PDXs (Tables S9 and S10) was also investigated. The MSCs were able to activate pathways driven by a number of ICAKs common to the two PDX types and also kinases that are specific to each type (Fig. 8C).

Considering the varied effects observed from co-culture with MSCs in different CCA cells, including both inhibition and enhancement of proliferation, the effects of activating such kinases were investigated. Those inactive in WD-PDXs, and with potential to be restored by the addition of MSCs, include kinases such as ERBB3, EGFR, ERBB2 and MET, the activation of which is associated with growth promotion (Jin, 2020; Yoshikawa et al., 2008; Dai et al., 2012), with ERBB2, EGFR and MET associated with the proliferative molecular subclass of CCA (Sia et al., 2013). In contrast, PD-PDX kinases with potential to be restored by the addition of MSCs include ACVR1C (ALK7), MAP3K14 and ROR1, which may be inhibitors of tumour growth (Li et al., 2023; Allen et al., 2017; Michael et al., 2019), and JAK1, involved in the STAT3 signalling pathway characteristic of the inflammatory molecular subclass of CCA (Sia et al., 2013).

Thus, the ability of cells derived from both types of PDX to form spheroids and the influence of addition of MSCs on their viability and growth in 3D culture was investigated.

### Assessment of iMSC effects on CCA growth in 3D models using PDXs

CCA cells derived from each of the PDXs were used to establish mono- and co-culture spheroid models. The ability of cells from the PDXs to form spheroids varied based on their histological group: WD-PDXs required iMSCs for supporting spheroid formation, whereas PD-PDXs readily formed spheroids in both mono- and co-cultures. Mono-culture CCA1, CCA7 and CCA11 (Fig. S2) spheroids were easily disintegrated with pipetting, but live/dead staining demonstrated good viability of cells within the loose clusters. In the co-culture setting, CCA1, CCA7 and CCA11 spheroids initially formed loose cell aggregates on day 0, which then transformed into compact spheroids by day 3, gradually expanding in size over time. The co-culture spheroids derived from these PDXs maintained their structural integrity, exhibited good viability based on live/dead staining, and, when cell viability was quantified using PrestoBlue, there was higher cell viability than in the mono-culture spheroids. In contrast, mono-culture CCA2, CCA4, CCA5 and CCA6 PDXs (Fig. S3) readily formed well-defined spheroids, which were viable based on live/dead staining, and their volume and viability, quantified by PrestoBlue, increased over time. However, when co-cultured with iMSCs, although these PDXs also formed stable, viable spheroids that grew over time, their volume and viability were lower than those of the mono-culture spheroids.

## DISCUSSION

The findings presented in this study highlight the heterogeneous role of MSCs within CCA tumours, with potential to either enhance or inhibit CCA cell growth. Furthermore, they offer insights into the intricate interplay between CCA cells and MSCs within a 3D spheroid culture system, demonstrating that addition of MSCs to such models can activate signalling pathways missing in patient xenografts owing to loss of a human stromal microenvironment. Effects of co-culture were observed across models employing both primary CCA, expanded as PDXs, as well as established cell lines, with insights into mechanisms at the molecular level provided by transcriptomic analysis.

Bioinformatic analysis of paired patient and PDX samples was used to identify pathways that are dysregulated in the PDXs, including in cancer cells within the xenografts, likely due to the absence of human stromal components that provide paracrine signals. Interestingly, one of the pathways dysregulated was MET, which, in spite of maintained expression in the PDX, was not active. This can be explained by the known low affinity of mouse HGF, compared to human HGF, for the human MET receptor; the receptor being differently phosphorylated in response to mouse HGF leads to aberrant signal transduction and reduced activation of the pathway (Ikebuchi et al., 2013). Other paracrine signals absent in the PDXs may be due to similar species disconnects. We further observed that the PDXs fall into two categories – PD and WD types, and although dysregulated pathways are shared between them, there are also pathways unique to each type. The signals that drive such pathways in patient tumours could be derived from multiple components of the TME. However, given the importance of MSCs in CCA, we investigated the effect of introducing MSCs into 3D spheroid models of CCA.

Spheroid formation and morphology varied between different cell lines, different PDXs and also between mono- and co-culture spheroids. Some cancer cells appeared to have an innate ability to form spheroids, while others (represented both amongst the cell lines and PDXs) formed spheroids more easily in the presence of iMSCs. Furthermore, in some cases, small compact spheroids were formed, which were lacking in distinct structures. In contrast, ductal-like structures were apparent in some spheroids, and, in the case of one of the cell lines, these were more prominent when iMSCs were incorporated. The increased compactness of spheroids and the presence of prominent ductal structures suggest changes in cell adhesion, cell-cell interactions and ECM deposition (Cuiffo and Karnoub, 2012; Yuan et al., 2023; Ghosh et al., 2020). This aligns with the broader concept of crosstalk between cancer cells and the surrounding stroma, which is known to fuel tumour progression and orchestrate dynamic ECM remodelling (Poornima et al., 2022; Ridge et al., 2017), as well as the upregulated expression of genes associated with ECM and ECM-receptor interaction in co-culture conditions in this study.

In spite of such changes, and consistent with other studies (Liang et al., 2021; Hass, 2020), proliferation of some cancer cells may not be enhanced by the presence of iMSCs and may even be inhibited. Such cancer cells may have acquired mutations that make them autonomous, for example with constitutively activated growth receptors (Du and Lovly, 2018), and thus they do not require paracrine signals or may be unable to provide signals required by MSCs to differentiate into activated CAFs (Shamai et al., 2019; Blache et al., 2019; Wang et al., 2021). The latter scenario is particularly interesting as it may be that additional stromal cell signals (e.g. from immune cells) are required in some cases (Kuzet and Gaggioli, 2016).

Our data suggest that paracrine signals provided by MSCs have the potential to activate both pro- and anti-proliferative signalling pathways. Examples of pro-proliferative pathways include those involving ERBB3, EGFR, ERBB2 and MET (Jin, 2020; Yoshikawa et al., 2008; Dai et al., 2012), which were shown to be inhibited in the WD-PDXs compared to patient tissues and activated in our MSC co-culture model; this potentially explains why addition of MSCs to these PDXs *ex vivo* enhanced spheroid formation and viability. Examples of potentially anti-proliferative pathways activated by paracrine signalling include those involving ACVR1C, MAP3K14 and ROR1, linked to inhibition of cancer growth including in the context of CCA (Li et al., 2023; Allen et al., 2017; Michael et al., 2019). These pathways were missing in the PD-PDXs, and *ex vivo* co-culture with MSCs inhibited this type of PDX. Of course, other signalling pathway such as RET and HK2, identified as being missing in the PD-PDXs and capable of being activated in MSC co-cultures, can be pro-proliferative, so ultimately the phenotypic outcome will be dependent on the balance of the signals received by the cells and its particular molecular make-up. In this respect, it is interesting that our two groups of PDXs, which appear to respond differently to paracrine signals from MSCs, bear some relationship to the molecular subtypes of CCA previously described, with ERBB2, EGFR and MET considered markers of the proliferative molecular subclass of CCA and JAK1, which is part of the STAT3 signalling pathway, associated with the inflammatory molecular subclass of CCA (Sia et al., 2013). In the future, it would be of interest to investigate associations with other molecular markers linked to poor therapeutic responses and outcomes, which include mutations in drivers of proliferative signalling pathways, such as KRAS, SMAD4 and FGFR2 and EPHA2, which may provide means for cancer cells to become independent of paracrine signalling (Wang et al., 2022; Boerner et al., 2021).

Our Kaplan–Meier analysis, which investigated the correlation between differentially expressed genes in the co-culture model and the overall survival of individuals with intrahepatic CCA, provides support for the potential clinical relevance of the developed models. *ANGPTL4*, *C16ORF45*, *VSTM2L*, *SERPINE2*, *CAPRIN2* and *SPOCD1* emerged as key players significantly linked to overall survival, with elevated expression levels of these genes notably associated with unfavourable outcomes. Although additional investigation is necessary to understand, for example, whether they are expressed in the cancer cells or in the stromal cells, our analysis demonstrates that expression levels are dependent on co-culture and provides insights into specific genes, such as *SERPINE2*, known for its involvement in ECM dynamics. In pancreatic cancer, *SERPINE2* overexpression has been associated with significantly increased local invasiveness, accompanied by a substantial increase in ECM production (Buchholz et al., 2003). Furthermore, the observed association of *CAPRIN2* with cell cycle processes suggests a potential impact on cancer cell proliferation (Ai et al., 2020). Understanding the role of these genes can contribute to explaining the results of our models and establishing connections with clinical data.

Thus, this 3D co-culture model system provides potential for further investigation, including through more detailed analysis of signalling pathways activated in individual cells and PDXs, as well as building the complexity by addition of further cell types.

Characterisation of models is crucial if they are to be effectively used in drug development. It is important not only to use models that represent the spectrum of molecular changes that occur in the cancer cells themselves but also to incorporate relevant aspects of the TME. In the absence of paracrine signals from stromal cells, pathways that are active in patients may be missed when searching for relevant drug targets, or potentially useful drugs might be discarded because the relevant pathway is missing in the model. The co-culture system described in this paper could serve as an invaluable platform for further interrogating the complex signalling mechanisms orchestrating tumour-stroma interactions, and for use in drug development.

## MATERIALS AND METHODS

### Cell culture conditions

Wild-type KKU-M055, KKU-M213 and RBE cell lines were obtained from Professor John Gordan at the University of California under a Material Transfer Agreement and subjected to STR profiling. iMSCs were obtained through a collaboration with Dr James Dixon from the School of Pharmacy at the University of Nottingham; they were immortalised by overexpression of TERT (Matta et al., 2019). The CCA cell lines and iMSCs were cultured in high-glucose Dulbecco's modified essential medium (DMEM) supplemented with 1 mM L-glutamine and 10% heat-inactivated foetal bovine serum (10% DMEM) and were used below passage 40. All cell lines were incubated in a 5% CO_2_ air-humidified atmosphere at 37°C. Regular mycoplasma tests were conducted monthly to confirm the absence of contamination in the cell cultures. Luminescent cell lines were created by lentiviral transduction of CCA cells with a pLVX-Puro Vector containing Firefly luciferase and a Puromycin resistance gene (Clontech, Takara Bio Company, Otsu, Japan).

### Establishment of spheroid cultures

Cells were detached and resuspended in 10 ml pre-warmed 10% DMEM. A concentration of 5000 cells/ml was added as a mono- or co-culture to each well in a 96-well ultra-low attachment (ULA) round-bottom plate (Sigma-Aldrich, CLS3474). For the co-culture, cancer cells were co-cultured with iMSCs at a 1:2 ratio (cancer cells:MSCs). Mouse-derived basement membrane extract (BME) from Cultrex PathClear (Bio-techne, 3432-005-01) was thawed overnight on ice at 4°C and added at a concentration of 100 µg/ml. A volume of 200 µl (equivalent to 1000 cells) was added. The plate was centrifuged at 300 ***g*** for 10 min and then incubated in a 5% CO_2_ air-humidified atmosphere at 37°C.

### PDX spheroids

Tumours (CCA1, CCA2, CCA4, CCA5, CCA6, CCA7 and CCA11) were obtained as fresh surgical material from tumour resections at Nottingham University Hospitals NHS Trust, collected with informed patient consent and National Research Ethics Service (NRES) approval (NRES REC 10/H0405/6) and used in accordance with NRES approval (NRES REC 08/H0403/37). Immunodeficient female Rag2^−/−^ γC^−/−^ (Rag2G; 8-10 weeks) bred in-house under Home Office Project Licence P375A76F were used in this project; as only small numbers of mice were required for each initiation, we used what was available from our small in-house colony, which happened to be females, rather than custom breed mixed cohorts for this project, generating excess animals in the process. PDXs were generated by implantation of minced tumour fragments admixed with Matrigel (BD Biosciences) using an implant trochar (VetTech, UK) subcutaneously under local anaesthetic (EMLA Cream, Aspen Pharma, Ireland) in the mice under Home Office Project Licence 3003444, having been approved by the University of Nottingham Animal Welfare and Ethical Review Body. Mice were maintained in individually ventilated cages (IVCs) (Tecniplast, UK) within a barriered unit illuminated by fluorescent lights set to give a 12 h light-dark cycle, as recommended in the UK Home Office Animals (Scientific Procedures) Act 1986. The room was air conditioned by a system designed to maintain an air temperature range of 21±2°C and a humidity of 55±10%. Mice were housed in social groups during the procedure and provided with irradiated bedding and autoclaved nesting materials and environmental enrichment (Datesand, UK). Sterile irradiated 5V5R rodent diet (IPS Ltd, UK) and irradiated water (SLS, UK) was offered *ad libitum*. National Cancer Research Institute (NCRI) guidelines for the welfare and use of animals in cancer research, Laboratory Animal Science Association good practice guidelines and Federation of European Laboratory Animal Science Associations working group on pain and distress guidelines were also followed, as were the Animal Research: Reporting of *In Vivo* Experiments guidelines on the reporting of *in vivo* experiments. Tumours were measured weekly using Vernier calipers, and the volumes were calculated using the formula
(Eqn1)

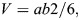
where *a* is the length and *b* is the width. Mice were also weighed weekly and given a daily health check by an experienced technician. Upon reaching maximum growth as allowed under the NCRI guidelines (mean diameter 1.2 cm), mice were killed by a Schedule 1 method (cervical dislocation), tumours were removed under aseptic conditions, and, during PDX passage, tissue was obtained and cells were isolated for use in spheroid models, using protocols previously established in our laboratory (Onion et al., 2016).

The cells were manually counted using a Neubauer chamber, and 1000 CCA cells were seeded for the spheroid mono-culture. For the spheroid co-culture, CCA cells were mixed with iMSCs at a 1:2 ratio (cancer cells:iMSCs). The cells, either alone or mixed, were seeded in ULA 96-well plates at a total volume of 200 µl per well. BME was added at a final concentration of 300 µg/ml. After centrifugation at 300 ***g*** for 10 min, the plates were placed in an incubator, maintaining a 5% CO_2_ air-humidified atmosphere at 37°C.

### Live/dead cell staining in spheroid culture

Live/dead staining was performed using a commercially available and pre-optimised kit (Thermo Fisher Scientific). Spheroids were washed with pre-warmed PBS and incubated with a solution containing 40 μM EthD-1 and 20 μM calcein acetoxymethyl ester. Cell imaging was performed using a fluorescence microscope (Nikon Eclipse Ti-U).

### Viability assays

Viability assays were conducted using PrestoBlue assay and the luciferase assay. For PrestoBlue assay, 100 µl of high-glucose DMEM was removed from each well and 10 µl PrestoBlue was added in each well. After incubation, the absorbance was measured using a microplate reader. For the luciferase assay, 10 µl D-luciferin (30 mg/ml) was added to each well, and the bioluminescence signal was measured using a luminometer reader.

### Histology

The spheroids were washed three times with PBS and fixed overnight at 4°C in 4% paraformaldehyde. After two further PBS washes, they were embedded in 2% agarose, fixed in 1× neutral buffer formalin and paraffin embedded to prepare spheroid microarrays according to a published protocol (Ivanov and Grabowska, 2017). For further characterisation of the spheroids, paraffin-embedded sections were prepared. H&E staining was performed to examine the morphology and internal structures of the spheroids. Immunohistochemical analysis was conducted to confirm the biliary phenotype using anti-CK7 (Dako, M7018; 1:100) and anti-CK19 (Dako, M0888; 1:100) as markers. Anti-vimentin (Dako, M0725; 1:100) was employed to localise iMSCs within the co-culture spheroids. Anti-Ki-67 (Dako, M7240; 1:100) was used to assess cell proliferation. Positive expression was quantified in five areas of each section by manually counting the positive cells and cancer cells and calculating the ratio of positively stained cancer cells over the total of cancer cells observed in each area.

### RNAseq

RNA extraction was performed using an AllPrep DNA/RNA/Protein Kit (Qiagen, 80004). Subsequently, RNAseq was carried out by Novogene (Cambridge, UK). The sequencing data underwent quality trimming and genome alignment (GRCh38). Integrated differential expression and pathway analysis (iDEP) was used to generate an expression matrix, filter the data and convert it to Ensemble gene IDs. Differential expression analysis was conducted using the DESeq2 package. Volcano plots were generated to visualise gene expression patterns. Pathway analysis was conducted using gene set enrichment analysis, using fold-change values obtained from DESeq2 results. The most significant hallmarks were selected and represented as KEGG-enrichment plots. Data were also analysed through the use of Qiagen IPA, in particular the canonical pathways and upstream regulator modules (Krämer et al., 2013). Canonical pathways analysis identified the pathways from the Qiagen IPA library of canonical pathways that were most significantly dysregulated, and Upstream Regulator Analysis identified upstream molecules predicted to be driving these changes. The significance of the association between the dataset and the canonical pathway/upstream regulator was measured by calculating a *P*-value determining the probability that the association between the genes in the dataset and the canonical pathway is explained by chance alone, and by calculation of a *z*-score to indicate the likelihood of activation or inhibition of that pathway. A *z*-score greater or less than 2 was considered significant. Heatmaps were drawn using Morpheus, and Venn diagrams were prepared using Venny.

### Image analysis

Fiji software, an enhanced distribution of ImageJ, was used to measure the area of spheroids.

### Analysis

Statistical analysis was performed using GraphPad Prism Version 9.4.1. For the 3D luciferase assays, the difference in cell viability between mono-culture and different co-cultures on each individual day was analysed using paired two-tailed Student's *t*-tests. Data are represented as mean±s.e.m. A significance level of *P*<0.05 was considered statistically significant. Cox proportional regression analysis for multiple genes and Kaplan–Meier analysis were carried out using the R2 Genomics Analysis and Visualization Platform, and statistical significance was tested using the log-rank test with Bonferroni correction to allow for multiple testing.

## Supplementary Material

10.1242/dmm.050716_sup1 Supplementary information

Table S2. List of upstream regulators identified through Ingenuity Pathway Analysis (IPA) analysis, including growth factors (GF), cytokines, kinases, and transcription regulators (TR).

Table S7. Differentially expressed genes identified by DESeq2 in KKU-M213-derived spheroids (monoculture vs co-culture). Gene ID, name, adjusted p-value, and log2-fold change are provided for each gene.
