# Supplementary material for: The role of mesenchymal cells in cholangiocarcinoma
Source: Dis Model Mech. 2024 Dec 13;17(12):dmm050716. doi: 10.1242/dmm.050716 (PMC11655028; doi:10.1242/dmm.050716)
Supplement: Supplementary information [file dmm-17-050716-s1.pdf]

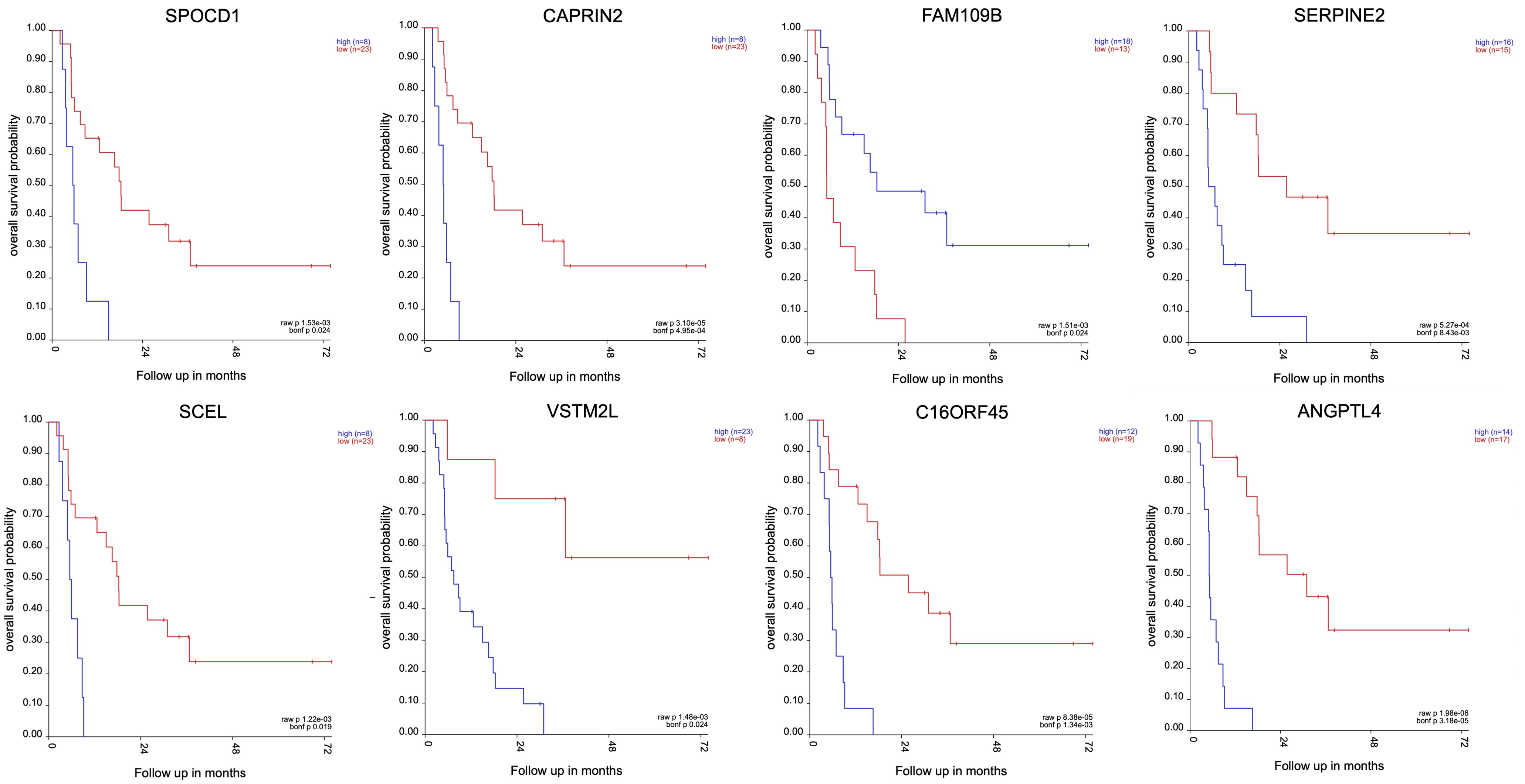

Fig. S1. Kaplan–Meier survival analysis of genes differentially expressed in co-culture model. Kaplan-Meier survival analysis was performed to assess the impact of gene expression on overall survival using dataset GSE89749 (33). The analysis focused on a subset of samples from patients with anatomic subtype, intrahepatic location, and fluke positive status to match the characteristics of KKU-M213.

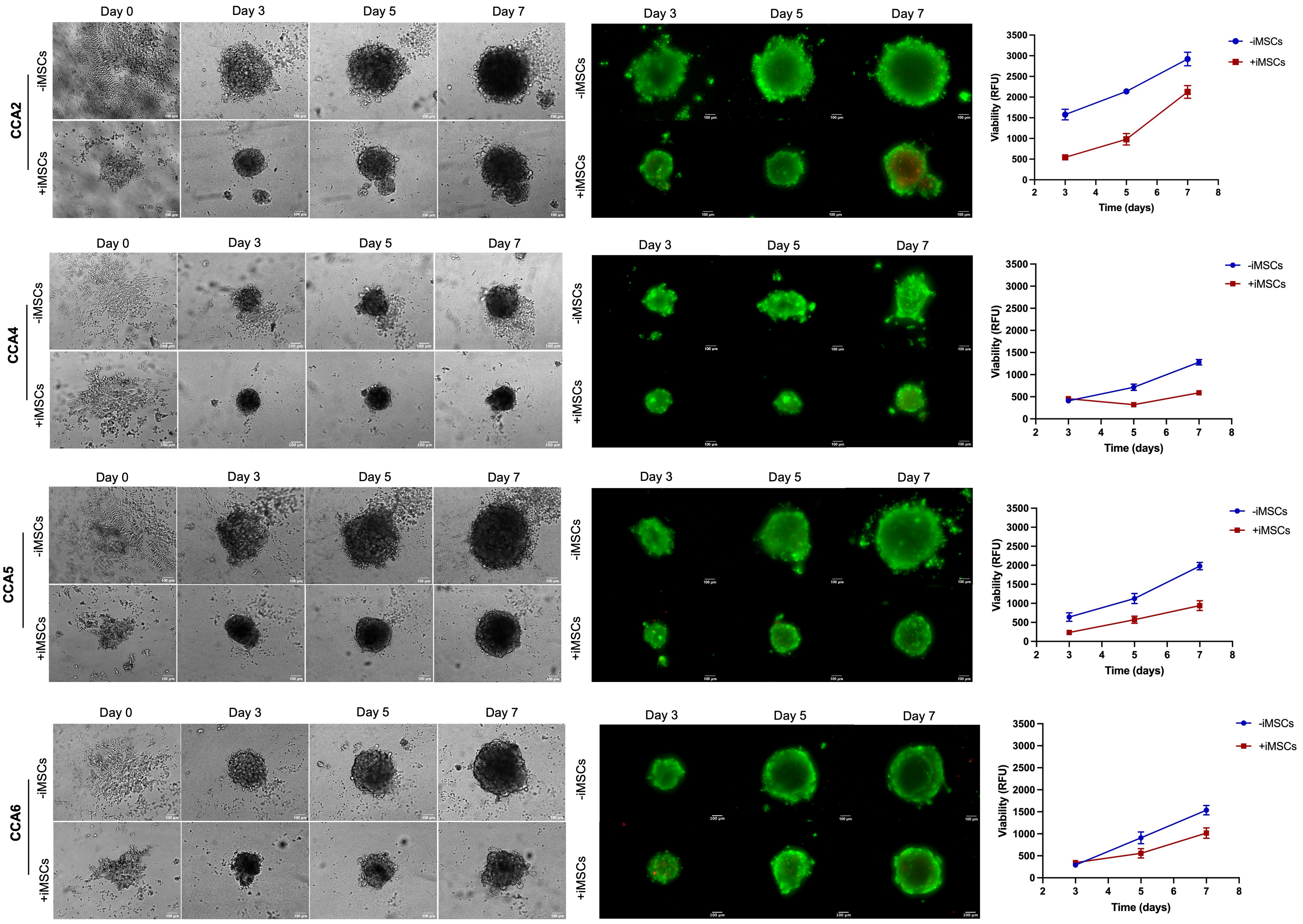

Fig. S2. Poorly-differentiated PDX-derived spheroids as monocultures and iMSCs co-cultures. CCA tissues (CCA2 (A), CCA4 (B), CCA5 (C) and CCA6 (D)) were enzymatically dissociated, and the cells were mono- or iMSCs co-cultured at 1:2 ratio (cancer cells:iMSCs) into an ULA 96-well round bottom plate at a final cell density of 1000 cells per well with 300μg/mL of basement membrane extract (BME). The effect of co-culture with iMSCs was assessed using brightfield microscopy at day 0, 3, 5 and 7 at 10x magnification. Live/dead cell staining was carried out using Calcein acetoxymethyl (AM) (green - live cells staining) and EthD-1 (red - dead cells staining) at days 3, 5 and 7. Representative spheroids are shown. Scale bar 100μm. Viability was monitored by PrestoBlue at day 3, 5 and 7 (Mean ± SEM).

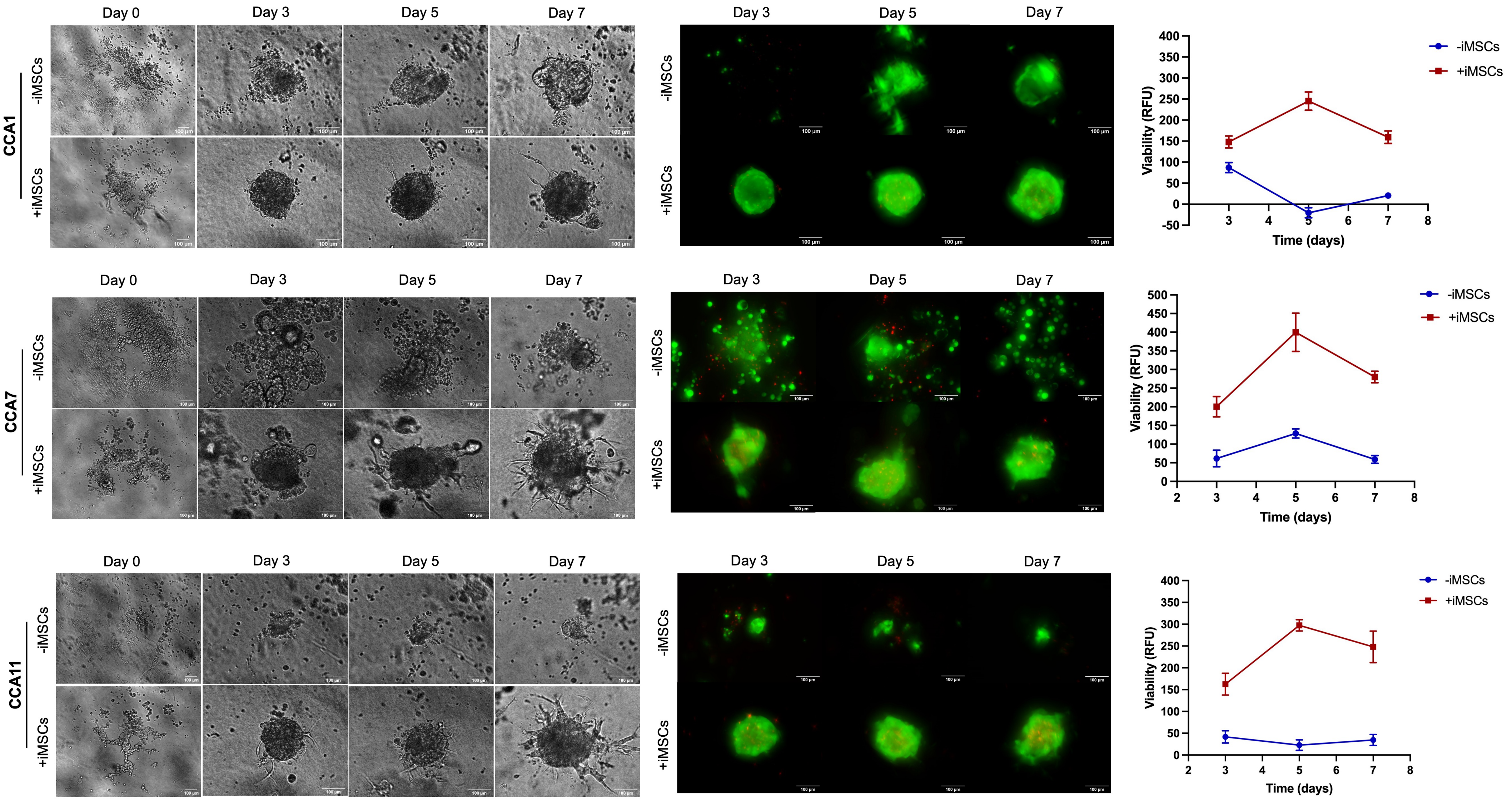

Fig. S3. Well-differentiated PDX-derived spheroids as monocultures and iMSCs co-cultures. CCA tissues (CCA1 (A), CCA7 (B) and CCA11 (C)) were enzymatically dissociated, and the cells were mono- or iMSCs co-cultured at 1:2 ratio (cancer cells:iMSCs) into an ULA 96-well round bottom plate at a final cell density of 1000 cells per well with 300µg/mL of basement membrane extract (BME). The effect of co-culture with iMSCs was assessed using brightfield microscopy at day 0, 3, 5 and 7 at 10x magnification. Live/dead cell staining was carried out using Calcein acetoxymethyl (AM) (green - live cells staining) and EthD-1 (red - dead cells staining) at days 3, 5 and 7. Representative spheroids are shown. Scale bar 100µm.

Table S1. Canonical pathways associated with cancer that were significantly dysregulated comparing patient tissues to PDXs.

Ingenuity Canonical Pathways	-log(p-value)	Ratio	z-score
S100 Family Signaling Pathway	14.9	0.28	9.54
FAK Signaling	11.7	0.27	9.70
PD-1, PD-L1 cancer immunotherapy pathway	8.25	0.40	-3.53
Tumor Microenvironment Pathway	6.39	0.31	5.98
Molecular Mechanisms of Cancer	6.38	0.22	9.99
Breast Cancer Regulation by Stathmin1	4.74	0.23	8.63
Cachexia Signaling Pathway	4.5	0.24	4.57
HOTAIR Regulatory Pathway	2.43	0.24	2.83
Role of Tissue Factor in Cancer	2.34	0.22	6.03
SPINK1 Pancreatic Cancer Pathway	2.31	0.33	-3.05
ID1 Signaling Pathway	1.71	0.21	2.60
Colorectal Cancer Metastasis Signaling	1.7	0.20	5.28
PTEN Signaling	1.53	0.21	-2.68
BEX2 Signaling Pathway	1.46	0.24	2.00
Thyroid Cancer Signaling	1.45	0.24	3.15
PCP (Planar Cell Polarity) Pathway	1.42	0.25	2.31
HEY1 Signaling Pathway	1.3	0.21	2.41

Table S2. List of upstream regulators identified through Ingenuity Pathway Analysis (IPA) analysis, including growth factors (GF), cytokines, kinases, and transcription regulators (TR).

Available for download at
https://journals.biologists.com/dmm/article-lookup/doi/10.1242/dmm.050716#supplementary-data

Disease Models & Mechanisms • Supplementary information

Table S3. Inhibited cancer-associated kinases (ICAKs) identified in PDX models.

Upstream Regulator	Expr Log Ratio	Expression (PDX vs PT)	Molecule Type	Predicted Activation State	Activation z-score	p-value of overlap
EPHA2	-1.94	3.85	kinase	Activated	2.16	6.34E-05
MET	-1.88	3.69	kinase	Activated	3.19	3.60E-08
MST1R	-1.72	3.29	kinase	Activated	2.80	6.54E-03
MAP3K21	-1.66	3.16	kinase	Activated	2.00	2.26E-02
TYRO3	-1.52	2.87	kinase	Activated	2.41	3.72E-06
PRKCZ	-1.35	2.55	kinase	Activated	3.39	6.32E-04
EIF2AK2	-1.05	2.07	kinase	Activated	3.21	1.54E-02
RET	-0.97	1.95	kinase	Activated	2.28	2.15E-07
CHUK	-0.87	1.83	kinase	Activated	6.10	6.93E-19
CDK6	-0.82	1.76	kinase	Activated	2.24	7.59E-02
CAMK2D	-0.66	1.58	kinase	Activated	2.00	2.56E-02
CDK5	-0.62	1.53	kinase	Activated	2.76	1.11E-05
EPHB4	-0.47	1.38	kinase	Activated	3.19	2.77E-03
PTK2	-0.44	1.35	kinase	Activated	3.45	2.42E-07
PAK1	-0.31	1.24	kinase	Activated	2.21	2.55E-01
IRAK1	-0.31	1.24	kinase	Activated	2.36	4.68E-04
TBK1	-0.29	1.22	kinase	Activated	3.03	7.47E-04
PDK1	-0.27	1.20	kinase	Activated	4.23	6.01E-03
MAP2K3	-0.23	1.17	kinase	Activated	3.26	3.58E-03
MAP2K1	-0.18	1.13	kinase	Activated	3.51	1.29E-11
RIPK2	-0.11	1.08	kinase	Activated	5.39	1.54E-04
MAPK8	-0.08	1.05	kinase	Activated	4.22	2.22E-08
MAPK9	-0.03	1.02	kinase	Activated	2.90	1.33E-06
MAPK14	-0.02	1.01	kinase	Activated	4.48	1.51E-18
PRKACA	0.07	0.95	kinase	Activated	2.03	2.18E-04
HK2	0.09	0.94	kinase	Activated	2.24	1.52E-01
IKBKG	0.10	0.93	kinase	Activated	4.97	1.34E-13
SRC	0.10	0.93	kinase	Activated	2.29	1.94E-07
RPS6KA5	0.11	0.93	kinase	Activated	2.12	3.34E-04
MAPK11	0.12	0.92	kinase	Activated	2.81	1.25E-02
RAF1	0.15	0.90	kinase	Activated	2.94	3.33E-06
IPMK	0.16	0.90	kinase	Activated	4.12	7.18E-06
PRKCD	0.19	0.88	kinase	Activated	3.61	3.43E-08
TYK2	0.20	0.87	kinase	Activated	3.02	4.09E-08
CDK9	0.23	0.85	kinase	Activated	2.79	2.66E-09
AKT1	0.25	0.84	kinase	Activated	2.60	2.81E-18
MAPK3	0.26	0.84	kinase	Activated	4.07	3.93E-08
ROCK1	0.28	0.83	kinase	Activated	3.97	9.44E-08
IKBKB	0.31	0.80	kinase	Activated	6.44	1.25E-26
MAP3K14	0.37	0.78	kinase	Activated	3.12	1.68E-07
DYRK1A	0.39	0.77	kinase	Activated	2.31	1.18E-05
PRKCE	0.46	0.72	kinase	Activated	3.60	1.37E-07
MAP3K1	0.49	0.71	kinase	Activated	2.81	1.35E-04
MKNK1	0.65	0.64	kinase	Activated	4.80	5.93E-05
ROR1	0.77	0.58	kinase	Activated	3.20	3.20E-03
CAMK4	0.79	0.58	kinase	Activated	2.77	1.77E-03

Disease Models & Mechanisms • Supplementary information

Table S4. Upstream regulators and inhibited cancer-associated kinases (ICAKs) identified in the well-differentiated group.

Upstream Regulator	Expr Log Ratio	Expression (PDX vs PT)	Molecule Type	Predicted Activation State	Activation z-score	p-value of overlap
**ACVR1C**	4.72	26.32	kinase	Inhibited	2.43	9.12E-02
**RET**	1.91	3.76	kinase	Inhibited	3.13	2.15E-07
**HK2**	1.71	3.27	kinase	Inhibited	2.00	3.72E-01
**TYRO3**	1.65	3.13	kinase	Inhibited	2.20	2.79E-03
**CAMK2D**	1.38	2.61	kinase	Inhibited	2.00	4.02E-02
**CHUK**	1.29	2.44	kinase	Inhibited	5.13	5.97E-15
**PRKCZ**	1.24	2.36	kinase	Inhibited	2.60	1.40E-02
**CAMK4**	1.06	2.09	kinase	Inhibited	3.23	1.24E-03
**RIPK2**	1.04	2.06	kinase	Inhibited	2.94	4.52E-05
**MAPK11**	0.94	1.92	kinase	Inhibited	2.16	7.27E-03
**CDK5**	0.90	1.87	kinase	Inhibited	2.27	2.13E-04
**PDK1**	0.76	1.69	kinase	Inhibited	3.13	2.10E-03
**PTK2**	0.71	1.64	kinase	Inhibited	3.17	1.25E-06
**IKBKG**	0.71	1.63	kinase	Inhibited	4.76	1.44E-11
**EPHB4**	0.59	1.51	kinase	Inhibited	2.85	6.43E-04
**PRKACA**	0.53	1.44	kinase	Inhibited	2.44	3.82E-05
**MAP2K1**	0.53	1.44	kinase	Inhibited	2.69	3.60E-14
**MAPK8**	0.49	1.41	kinase	Inhibited	2.87	8.35E-10
**PAK2**	0.48	1.39	kinase	Inhibited	2.14	5.61E-04
**IRAK1**	0.46	1.38	kinase	Inhibited	2.46	8.17E-03
**MAP3K14**	0.45	1.37	kinase	Inhibited	3.29	5.94E-06
**MAP2K3**	0.43	1.35	kinase	Inhibited	3.04	7.86E-04
**TYK2**	0.16	1.12	kinase	Inhibited	3.58	1.86E-05
**MAPK9**	0.11	1.08	kinase	Inhibited	3.11	8.74E-05
**ROCK1**	0.08	1.05	kinase	Inhibited	3.45	4.83E-07
**MAPK14**	0.07	1.05	kinase	Inhibited	3.86	3.85E-16
**AKT1**	0.05	1.04	kinase	Inhibited	2.50	1.31E-17
**IPMK**	0.05	1.04	kinase	Inhibited	3.18	2.86E-03
**PRKCD**	0.05	1.03	kinase	Inhibited	3.38	3.94E-09
**DYRK1A**	0.04	1.03	kinase	Inhibited	2.44	3.40E-03
**PRKCE**	-0.13	0.91	kinase	Inhibited	2.73	2.21E-04
**ROR1**	-0.19	0.88	kinase	Inhibited	2.20	6.10E-03
**FGFR2**	-0.34	0.79	kinase	Inhibited	2.31	2.45E-08
**MAP3K1**	-0.35	0.79	kinase	Inhibited	2.22	7.86E-04
**IRAK4**	-0.46	0.73	kinase	Inhibited	2.12	4.36E-03
**MAPK3**	-0.48	0.72	kinase	Inhibited	2.57	3.04E-05
**MAP3K3**	-0.62	0.65	kinase	Inhibited	2.39	5.41E-06
**IKBKB**	-0.64	0.64	kinase	Inhibited	5.93	1.52E-18
**MKNK1**	-0.67	0.63	kinase	Inhibited	2.44	6.20E-04
**NEK6**	-0.84	0.56	kinase	Inhibited	2.00	3.35E-02
**JAK1**	-0.92	0.53	kinase	Inhibited	2.68	1.24E-02

Disease Models & Mechanisms • Supplementary information

Table S5. Upstream regulators and inhibited cancer-associated kinases (ICAKs) identified in the poorly differentiated group.

Upstream Regulator	Expr Log Ratio	Expression (PDX vs PT)	Molecule Type	Predicted Activation State	Activation z-score	p-value of overlap
**ERBB3**	2.67	6.38	kinase	Inhibited	2.154	5.73E-13
**MST1R**	2.21	4.62	kinase	Inhibited	2.449	3.81E-03
**MAP3K21**	2.15	4.45	kinase	Inhibited	2	1.46E-03
**EGFR**	2.14	4.41	kinase	Inhibited	2.213	5.51E-06
**ERBB2**	2.11	4.33	kinase	Inhibited	3.731	1.01E-11
**PRKCZ**	1.57	2.97	kinase	Inhibited	2.774	1.75E-03
**TYRO3**	1.44	2.71	kinase	Inhibited	2.412	2.30E-06
**PLK4**	1.16	2.24	kinase	Inhibited	2.449	5.06E-03
**MET**	1.16	2.24	kinase	Inhibited	4.548	2.48E-06
**BMPR1A**	0.90	1.86	kinase	Inhibited	2.207	1.31E-02
**PIK3CB**	0.75	1.68	kinase	Inhibited	2.216	4.11E-04
**SRC**	0.46	1.38	kinase	Inhibited	2.619	8.84E-10
**PRKCA**	0.42	1.34	kinase	Inhibited	2.674	1.09E-03
**CHUK**	0.40	1.32	kinase	Inhibited	5.426	3.62E-15
**EPHB4**	0.39	1.31	kinase	Inhibited	2.764	4.37E-03
**IKBKB**	0.32	1.25	kinase	Inhibited	5.692	3.33E-22
**IRAK1**	0.19	1.14	kinase	Inhibited	2.036	1.03E-03
**PTK2**	0.16	1.12	kinase	Inhibited	3.063	9.14E-09
**MAPK3**	0.15	1.11	kinase	Inhibited	2.742	1.67E-06
**TBK1**	0.05	1.03	kinase	Inhibited	3.575	1.67E-06
**MAP2K3**	0.04	1.02	kinase	Inhibited	2.95	1.28E-04
**MAPK14**	0.03	1.02	kinase	Inhibited	4.766	3.86E-18
**RAF1**	-0.05	0.97	kinase	Inhibited	3.985	1.27E-02
**RIPK3**	-0.07	0.95	kinase	Inhibited	2.4	2.01E-03
**MAP2K4**	-0.14	0.91	kinase	Inhibited	2.8	4.73E-02
**MAP2K1**	-0.20	0.87	kinase	Inhibited	4.861	6.11E-11
**CAMK2D**	-0.25	0.84	kinase	Inhibited	2	1.05E-02
**PDK1**	-0.29	0.82	kinase	Inhibited	3.162	3.84E-02
**IPMK**	-0.35	0.78	kinase	Inhibited	3.742	4.75E-05

Disease Models & Mechanisms • Supplementary information

**MAPK8**	-0.42	0.75	kinase	Inhibited	2.244	2.47E-06
**PRKCD**	-0.43	0.74	kinase	Inhibited	2.78	9.99E-03
**BRD4**	-0.44	0.74	kinase	Inhibited	4.838	8.29E-09
**MAP2K6**	-0.52	0.70	kinase	Inhibited	2.183	1.30E-08
**MKNK1**	-0.53	0.69	kinase	Inhibited	4.214	5.90E-05
**AKT1**	-0.56	0.68	kinase	Inhibited	4.292	1.15E-12
**MAP3K1**	-0.59	0.67	kinase	Inhibited	2.477	4.20E-04
**TYK2**	-0.61	0.65	kinase	Inhibited	2.688	2.08E-06
**CDK9**	-0.62	0.65	kinase	Inhibited	2.415	2.95E-07
**STK40**	-0.63	0.65	kinase	Inhibited	3.138	7.79E-08
**ROCK1**	-0.64	0.64	kinase	Inhibited	3.719	3.41E-09
**BMPR2**	-0.81	0.57	kinase	Inhibited	2.413	2.62E-02
**PLK2**	-0.83	0.56	kinase	Inhibited	2.236	2.30E-02
**PRKCE**	-0.84	0.56	kinase	Inhibited	3.264	1.13E-02
**IKBKG**	-0.88	0.54	kinase	Inhibited	4.302	9.31E-07
**JAK2**	-0.88	0.54	kinase	Inhibited	2.201	8.79E-08

Disease Models & Mechanisms • Supplementary information

Table S6. Genes differentially expressed in the co-culture model associated with overall survival. The adjusted p-values were derived from Kaplan-Meier survival analysis using Cox proportional regression. Additionally, the log2 difference in expression in the co-culture is provided for each gene.

Gene Symbol	Adjusted p-value	Log2 Fold Change in Expression in Co-culture
**SPOCD1**	0.024	2.241128
**CAPRIN2**	4.95E-04	1.233663
**FAM109B**	0.024	9.388593
**SERPINE2**	8.43E-03	2.074787
**SCEL**	0.019	-1.55945
**VSTM2L**	0.024	6.567292
**C16ORF45**	1.30E-03	6.774376
**ANGPTL4**	3.20E-05	1.230297

Table S7. Differentially expressed genes identified by DESeq2 in KKU-M213-derived spheroids (monoculture vs co-culture). Gene ID, name, adjusted p-value, and log2-fold change are provided for each gene.

Available for download at
https://journals.biologists.com/dmm/article-lookup/doi/10.1242/dmm.050716#supplementary-data

Table S8. Inhibited cancer-associated kinases (ICAKs) activated in MSC co-cultures identified from whole PDX analysis.

Upstream Regulator	Expr Log Ratio	Expression (PDX vs PT)	Molecule Type	Predicted Activation State	Activation z-score	p-value of overlap
**EPHA2**	-		-	-	-	-
**MET**	0.01	1.00	kinase	Activated	3.39	2.68E-06
**MST1R**	-		-	-	-	-
**MAP3K21**	-0.27	0.83	kinase			6.63E-03
**TYRO3**	-		-	-	-	-
**PRKCZ**	-0.18	0.88	kinase		1.98	4.50E-02
**EIF2AK2**	-		-	-	-	-
**RET**			kinase	Activated	2.39	3.33E-05
**CHUK**	-0.01	0.99	kinase	Activated	3.06	2.57E-08
**CDK6**	-0.13	0.91	kinase		1.89	1.72E-01
**CAMK2D**	-		-	-	-	-
**CDK5**	-		-	-	-	-
**EPHB4**	-		-	-	-	-
**PTK2**	0.05	1.04	kinase	Activated	2.39	9.84E-08
**PAK1**	-		-	-	-	-
**IRAK1**	0.09	1.06	kinase		0.72	3.02E-02
**TBK1**	-		-	-	-	-
**PDK1**	-		-	-	-	-
**MAP2K3**	0.08	1.05	kinase		1.98	3.86E-06
**MAP2K1**	0.01	1.01	kinase	Activated	2.30	7.77E-07
**RIPK2**	-		-	-	-	-
**MAPK8**	-0.15	0.90	kinase		0.96	1.41E-04
**MAPK9**	-		-	-	-	-
**MAPK14**	0.14	1.10	kinase		1.71	3.27E-04
**PRKACA**	-		-	-	-	-
**HK2**	-0.82	0.57	kinase	Activated	2.22	1.06E-03
**IKBKG**	0.04	1.03	kinase	Activated	2.68	7.10E-06
**SRC**	0.08	1.05	kinase		1.41	1.58E-06
**RPS6KA5**	-		-	-	-	-

Disease Models & Mechanisms • Supplementary information

**MAPK11**	-		-	-	-	-
**RAF1**	-		-	-	-	-
**IPMK**	-0.26	0.84	kinase		1.22	7.53E-03
**PRKCD**	-0.19	0.88	kinase	Activated	2.47	7.00E-04
**TYK2**	-		-	-	-	-
**CDK9**	0.06	1.04	kinase			8.52E-03
**AKT1**	0.46	1.38	kinase	Activated	3.03	1.44E-06
**MAPK3**	-		-	-	-	-
**ROCK1**	0.20	1.15	kinase	Activated	3.44	1.98E-09
**IKBKB**	0.14	1.10	kinase	Activated	3.29	3.05E-10
**MAP3K14**	0.17	1.12	kinase	Activated	2.56	1.95E-04
**DYRK1A**	-		-	-	-	-
**PRKCE**	-0.22	0.86	kinase	Activated	2.60	1.05E-02
**MAP3K1**	-0.28	0.82	kinase	Activated	2.00	8.52E-03
**MKNK1**	-0.12	0.92	kinase	Activated	2.16	2.71E-03
**ROR1**	0.27	1.20	kinase	Activated	2.76	1.58E-03
**CAMK4**	-		-	-	-	-

Disease Models & Mechanisms • Supplementary information

Table S9. Inhibited cancer-associated kinases (ICAKs) activated in MSC co-cultures identified from well-differentiated PDXs.

Upstream Regulator	Expr Log Ratio	Expression (PDX vs PT)	Molecule Type	Predicted Activation State	Activation z-score	p-value of overlap
**ERBB3**	-1.07		kinase	Activated	2.10	7.66E-11
**MST1R**	-	1.00	-	-	-	-
**MAP3K21**	-0.27		kinase			6.63E-03
**EGFR**	-0.10	0.83	kinase	Activated	2.73	9.69E-06
**ERBB2**	-0.29		kinase	Activated	2.75	1.92E-21
**PRKCZ**	-0.18	0.88	kinase		1.98	4.50E-02
**TYRO3**	-		-	-	-	-
**PLK4**	-		-	-	-	-
**MET**	0.01	0.99	kinase	Activated	3.39	2.68E-06
**BMPR1A**	-0.17	0.91	kinase		0.56	1.55E-03
**PIK3CB**	-		-	-	-	-
**SRC**	0.08		kinase		1.41	1.58E-06
**PRKCA**	-		-	-	-	-
**CHUK**	-0.01	1.04	kinase	Activated	3.06	2.57E-08
**EPHB4**	-		-	-	-	-
**IKBKB**	0.14	1.06	kinase	Activated	3.29	3.05E-10
**IRAK1**	0.09		kinase		0.72	3.02E-02
**PTK2**	0.05		kinase	Activated	2.39	9.84E-08
**MAPK3**	-	1.05	-	-	-	-
**TBK1**	-	1.01	-	-	-	-
**MAP2K3**	0.08		kinase		1.98	3.86E-06
**MAPK14**	0.14	0.90	kinase		1.71	3.27E-04
**RAF1**	-		-	-	-	-
**RIPK3**	0.15	1.10	kinase			4.10E-02
**MAP2K4**	-		-	-	-	-
**MAP2K1**	0.01	0.57	kinase	Activated	2.30	7.77E-07
**CAMK2D**	-	1.03	-	-	-	-
**PDK1**	-	1.05	-	-	-	-
**IPMK**	-0.26		kinase		1.22	7.53E-03

Disease Models & Mechanisms • Supplementary information

**MAPK8**	-0.15		kinase		0.96	1.41E-04
**PRKCD**	-0.19		kinase	Activated	2.47	7.00E-04
**BRD4**		0.84	kinase	Activated	3.50	7.45E-07
**MAP2K6**	-0.79	0.88	kinase		1.69	3.20E-04
**MKNK1**	-0.12		kinase	Activated	2.16	2.71E-03
**AKT1**	0.46	1.04	kinase	Activated	3.03	1.44E-06
**MAP3K1**	-0.28	1.38	kinase	Activated	2.00	8.52E-03
**TYK2**	-		-	-	-	-
**CDK9**	0.06	1.15	kinase			8.52E-03
**STK40**	-0.06	1.10	kinase		1.16	2.71E-04
**ROCK1**	0.20	1.12	kinase	Activated	3.44	1.98E-09
**BMPR2**	1.70		kinase		1.13	3.87E-02
**PLK2**	-	0.86	-	-	-	-
**PRKCE**	-0.22	0.82	kinase	Activated	2.60	1.05E-02
**IKBKG**	0.04	0.92	kinase	Activated	2.68	7.10E-06
**JAK2**	0.35	1.20	kinase		1.73	3.65E-03

Table S10. Inhibited cancer-associated kinases (ICAKs) activated in MSC co-cultures identified from poorly-differentiated PDXs.

Upstream Regulator	Expr Log Ratio	Expression (PDX vs PT)	Molecule Type	Predicted Activation State	Activation z-score	p-value of overlap
**ACVR1C**	-1.32		kinase	Activated	2.61	2.94E-04
**RET**		1.00	kinase	Activated	2.39	3.33E-05
**HK2**	-0.82		kinase	Activated	2.22	1.06E-03
**TYRO3**	-	0.83	-	-	-	-
**CAMK2D**	-		-	-	-	-
**CHUK**	-0.01	0.88	kinase	Activated	3.06	2.57E-08
**PRKCZ**	-0.18		kinase		1.98	4.50E-02
**CAMK4**	-		-	-	-	-
**RIPK2**	-	0.99	-	-	-	-
**MAPK11**	-	0.91	-	-	-	-
**CDK5**	-		-	-	-	-
**PDK1**	-		-	-	-	-
**PTK2**	0.05		kinase	Activated	2.39	9.84E-08
**IKBKG**	0.04	1.04	kinase	Activated	2.68	7.10E-06
**EPHB4**	-		-	-	-	-
**PRKACA**	-	1.06	-	-	-	-
**MAP2K1**	0.01		kinase	Activated	2.30	7.77E-07
**MAPK8**	-0.15		kinase		0.96	1.41E-04
**PAK2**	-0.06	1.05	kinase		0.45	1.64E-02
**IRAK1**	0.09	1.01	kinase		0.72	3.02E-02
**MAP3K14**	0.17		kinase	Activated	2.56	1.95E-04
**MAP2K3**	0.08	0.90	kinase		1.98	3.86E-06
**TYK2**	-		-	-	-	-
**MAPK9**	-	1.10	-	-	-	-
**ROCK1**	0.20		kinase	Activated	3.44	1.98E-09
**MAPK14**	0.14	0.57	kinase		1.71	3.27E-04
**AKT1**	0.46	1.03	kinase	Activated	3.03	1.44E-06
**IPMK**	-0.26	1.05	kinase		1.22	7.53E-03
**PRKCD**	-0.19		kinase	Activated	2.47	7.00E-04

Disease Models & Mechanisms • Supplementary information

**DYRK1A**	-		-	-	-	-
**PRKCE**	-0.22		kinase	Activated	2.60	1.05E-02
**ROR1**	0.27	0.84	kinase	Activated	2.76	1.58E-03
**FGFR2**	-0.49	0.88	kinase		-0.08	4.77E-07
**MAP3K1**	-0.28		kinase	Activated	2.00	8.52E-03
**IRAK4**	-	1.04	-	-	-	-
**MAPK3**	-	1.38	-	-	-	-
**MAP3K3**	0.46		kinase			3.98E-03
**IKBKB**	0.14	1.15	kinase	Activated	3.29	3.05E-10
**MKNK1**	-0.12	1.10	kinase	Activated	2.16	2.71E-03
**NEK6**	-	1.12	-	-	-	-
**JAK1**	0.40		kinase	Activated	2.80	2.71E-03

Disease Models & Mechanisms • Supplementary information
